# Large-Scale Multiplexing Permits Full-Length Transcriptome Annotation of 32 Bovine Tissues From a Single Nanopore Flow Cell

**DOI:** 10.3389/fgene.2021.664260

**Published:** 2021-05-20

**Authors:** Michelle M. Halstead, Alma Islas-Trejo, Daniel E. Goszczynski, Juan F. Medrano, Huaijun Zhou, Pablo J. Ross

**Affiliations:** Department of Animal Science, University of California, Davis, Davis, CA, United States

**Keywords:** transcriptome, annotation, cattle, tissue-specific, alternative splicyng, long-read sequencing, full-length transcript

## Abstract

A comprehensive annotation of transcript isoforms in domesticated species is lacking. Especially considering that transcriptome complexity and splicing patterns are not well-conserved between species, this presents a substantial obstacle to genomic selection programs that seek to improve production, disease resistance, and reproduction. Recent advances in long-read sequencing technology have made it possible to directly extrapolate the structure of full-length transcripts without the need for transcript reconstruction. In this study, we demonstrate the power of long-read sequencing for transcriptome annotation by coupling Oxford Nanopore Technology (ONT) with large-scale multiplexing of 93 samples, comprising 32 tissues collected from adult male and female Hereford cattle. More than 30 million uniquely mapping full-length reads were obtained from a single ONT flow cell, and used to identify and characterize the expression dynamics of 99,044 transcript isoforms at 31,824 loci. Of these predicted transcripts, 21% exactly matched a reference transcript, and 61% were novel isoforms of reference genes, substantially increasing the ratio of transcript variants per gene, and suggesting that the complexity of the bovine transcriptome is comparable to that in humans. Over 7,000 transcript isoforms were extremely tissue-specific, and 61% of these were attributed to testis, which exhibited the most complex transcriptome of all interrogated tissues. Despite profiling over 30 tissues, transcription was only detected at about 60% of reference loci. Consequently, additional studies will be necessary to continue characterizing the bovine transcriptome in additional cell types, developmental stages, and physiological conditions. However, by here demonstrating the power of ONT sequencing coupled with large-scale multiplexing, the task of exhaustively annotating the bovine transcriptome – or any mammalian transcriptome – appears significantly more feasible.

## Introduction

The proteome diversity observed in eukaryotes is largely attributed to alternative transcript isoforms, which result from use of alternate transcription start sites, polyadenylation sites, and splice sites. In particular, the complexity of alternative splicing seems to have increased during the course of evolution (Keren et al., 2010), such that transcript isoforms exist for the majority of genes in higher order eukaryotes (Pan et al., 2008; Wang et al., 2008; Mercer et al., 2012). This diversification of the transcriptome and proteome not only drives adaptation and speciation (Harr and Turner, 2010; Mudge et al., 2011), but also facilitates cellular diversity and the development of complex organisms with tissues and organs (Graveley, 2001; Linker et al., 2019). Indeed, transcript isoforms and splicing patterns vary between cell types, tissues, developmental stages, and environmental conditions (Kalsotra et al., 2008; Wang et al., 2008; Vaquero-Garcia et al., 2016; Zhang et al., 2016). Moreover, because alternative splicing can fundamentally alter protein structure and function, aberrant isoforms have been linked to various diseases, including cancer (Paronetto et al., 2016; Zhang et al., 2019).

More than 90% of human genes are subject to alternative splicing (Pan et al., 2008; Workman et al., 2019); as such, considerable efforts have been made by consortia such as GENCODE to exhaustively annotate transcript isoforms in humans and mice. However, projects seeking to annotate the genomes of non-model organisms generally lack the necessary resources for manual curation. Consequently, transcriptome annotations for non-model organisms, including species of high economic significance like livestock, are often incomplete or inaccurate (Andersson et al., 2015; Ungaro et al., 2017). Moreover, transcriptome complexity and splicing patterns are not well-conserved between species (Barbosa-Morais et al., 2012). Transcript structures inferred from related species are therefore likely to be insufficient or inaccurate.

Worldwide, over a billion cattle (*Bos taurus*) are raised for meat and dairy production (Robinson et al., 2014), and although selection programs have significantly benefited from genomics tools in the past decade (Meredith et al., 2012; Saatchi et al., 2012; Thompson-Crispi et al., 2014; García-Ruiz et al., 2016), a comprehensive characterization of the bovine transcriptome is essential to improve our understanding of the biological processes that underpin complex traits like productivity, efficiency, and disease resistance (Georges et al., 2019).

Until recently, transcriptome annotations – including that of the bovine genome – were primarily based on short-read RNA-seq data from next-generation sequencing (NGS) platforms. The high throughput of these sequencers was optimal for quantifying gene expression, but because of sequencing length limitations, it is necessary to fragment RNA or cDNA during library preparation. The resulting reads are generally shorter (<200 bases) than most full-length transcripts, and although several computational approaches have been developed to reconstruct transcript structures from short-read RNA-seq data, they do not always infer the correct structures (Grabherr et al., 2011; Trapnell et al., 2012; Pertea et al., 2015; Conesa et al., 2016).

Alternatively, long-read sequencing technologies, such as Pacific Biosciences (PacBio) (McCarthy, 2010; Rhoads and Au, 2015) and Oxford Nanopore Technologies (ONT) (Bayega et al., 2018), have made it possible to sequence reads up to 50 kb in length, allowing for the sequencing of full-length transcripts without the need for reconstruction. In recent years, PacBio single-molecule real-time (SMRT) isoform sequencing (Iso-seq) has been implemented to improve transcriptome annotations in humans (Sharon et al., 2013; Tilgner et al., 2014), rabbits (Chen et al., 2017), chickens (Thomas et al., 2014; Kuo et al., 2017), pigs (Li et al., 2018; Beiki et al., 2019), and cattle (Rosen et al., 2020). Indeed, the transcriptome accompanying the most recent bovine genome assembly was curated from both short-read RNA-seq and Iso-seq data (Rosen et al., 2020); however, the Iso-seq dataset was limited, as it included fewer tissue transcriptomes than the short-read RNA-seq data, and was of considerably lower sequencing depth, producing only about a half a million consensus reads.

An alternative long-read sequencing technology, ONT sequencing, measures changes in ionic current as fragments move through protein nanopores, and does not depend on enzyme-based nucleotide incorporation or detection of fluorescence (Ip et al., 2015). Due to its affordability and higher throughput – the ONT PromethION generates 20 times more reads per flow cell than the PacBio Sequel II (Garalde et al., 2018) – ONT has been widely used for transcriptome annotation in organisms ranging from yeast to humans (Sharon et al., 2013; Tilgner et al., 2014; Oikonomopoulos et al., 2016; Byrne et al., 2017; Jenjaroenpun et al., 2018; Kadobianskyi et al., 2019; Seki et al., 2019; Sessegolo et al., 2019; Workman et al., 2019; Müller et al., 2020; Sahoo et al., 2020), permitting the discovery of isoforms that were difficult to observe from short-read sequencing alone (Steijger et al., 2013; Venturini et al., 2018).

Despite the incorporation of Iso-seq data (Rosen et al., 2020), the bovine transcriptome still only includes 1.59 transcripts per gene on average, whereas the human genome annotation accounts for an average of 3.78 transcript isoforms per gene (Ensembl v101 annotations). This discrepancy suggests that the transcriptomic complexity of the bovine genome has yet to be fully characterized, and that current annotations are likely missing information on rare and tissue-specific isoforms. In this study, we coupled ONT sequencing with large-scale multiplexing to identify and characterize the expression of transcript isoforms in cattle. From a single ONT flow cell, we obtained over 25 million full-length uniquely mapped reads, allowing us to characterize the transcriptomes of 32 adult bovine tissues across four individuals. This powerful approach paves the way for future transcriptomic studies, facilitating research on a wider variety of cell types, physiological conditions, and developmental stages. Moreover, the resulting transcript predictions will help to inform selection programs seeking to improve production traits, fertility, and environmental adaptation – factors which are of considerable scientific and economic interest.

## Materials and Methods

### Sample Collection

Tissue samples were collected from two male and two female Line 1 Hereford cattle, aged 14 months old, which were provided by the Fort Keogh Livestock and Range Research lab. Animals were euthanized by captive bolt under USDA inspection at the University of California, Davis, with all permissions obtained and in concordance with Protocol for Animal Care and Use no. 18464 (approved by Institutional Animal Care and Use Committee at the University of California, Davis). Samples were collected within 1–2 h of euthanasia, flash frozen in liquid nitrogen, and stored at –80°C until processing.

### RNA Extraction and Library Construction

Frozen tissues kept at –80°C were homogenized with a mortar and pestle in liquid nitrogen. Total RNA was extracted using Trizol (Invitrogen, Carlsbad, CA, United States) followed by a column clean-up using the Direct-zol RNA Mini Prep Plus kit (Zymo Research, Irvine, CA, United States) and performing an in-column DNA digestion. Integrity of the DNase-treated RNA was verified on the Experion electrophoresis system (Bio-Rad, Hercules, CA, United States). For each sample, 50 ng total RNA was transferred to 0.2 ml PCR tubes and adjusted to a final volume of 9 μl with nuclease free water. Reactions were prepared (9 μl total RNA, 1 μl 10 μM VNP primer, 1 μl 10 mM dNTPs) and incubated for 5 min at 65°C, then snap cooled on a pre-chilled freezer block. Strand-switching buffer (4 μl 5x RT buffer, 1 μl RNaseOUT, 1 μl nuclease-free water, and 2 μl 10 μM strand-switching primer) was then added to the snap-cooled, annealed mRNA, and incubated at 42°C for 2 min. One μl of Maxima H Minus Reverse Transcriptase was added, and reactions were incubated at 42°C for 90 min, 85°C for 5 min, then held at 4°C. A round of PCR was used to introduce barcodes to the cDNA using the Oxford Nanopore PCR barcoding expansion 1-96 kit (Cat. No. EXP-PBC096). Barcoding PCR reactions were set up for each cDNA (1 μl PCR barcode, 19 μl first-strand cDNA, 20 μl LongAmp Taq 2x master mix), and cycled for [3 min at 95°C] x1 cycle, [15 s at 95°C, 15 s at 62°C, 7 min at 65°C] x13 cycles, [15 min at 65°C] x1 cycle, then held at 4°C. Each barcoded cDNA was purified in 1x Ampure XP Beads, eluted in 20 μl of nuclease free water and quantified using Qubit. Barcoded cDNAs were pooled in a final volume of 47 μl. The DNA Technologies Core and Expression Analysis Laboratory at the University of California Davis performed adapter ligation on the cDNA pool with the SQK-DCS109 kit following manufacturer’s guidelines. Finally, 50 fmol of adapter ligated library was loaded onto a PromethION flow cell (vR9.4.1).

### Pre-processing of ONT Sequencing Data

The quality of raw sequencing data, including read length and average quality, was checked using Nanoplot (v1.0.0). Base calling and demultiplexing (Supplementary Table 1) were performed using ont-guppy-for-minknow (v3.0.5) and reads with a quality score below 7 were discarded. Data were then processed with Pychopper (v2.4.0) to identify and orient full-length reads; these were then mapped to the ARS-UCD1.2 genome assembly using minimap2 (v2.16r922) (Li, 2018) with options “-ax splice -uf -k14 -G 1000000.” The maximum allowable intron size was increased to 1 Mb, based on the longest intron observed in the Ensembl (v101) annotation. Uniquely mapped reads with a minimum quality score of 10 were extracted with Samtools (v1.7).

### Preliminary Analysis of Gene Expression

Uniquely mapped reads were used to obtain raw gene expression counts, based on the Ensembl v101 annotations for each species, using HTSeq (v0.11.2) (Anders et al., 2015) with options “-i gene_id –type = exon –stranded = yes –mode = intersection-non-empty.” Raw gene counts were subjected to variance stabilizing transformation (VST) with DESeq2 (v1.26.0) (Love et al., 2014) for principal components analysis, conducted with the prcomp function from the R package Stats (v3.6.3). Expression profiles of the top 5,000 genes with the most variance in VST counts were visualized with pheatmap (v1.0.12).

### Predicting Transcript Isoforms

Uniquely mapped reads from all samples were pooled to predict transcripts using the Pinfish pipeline (v0.1.0)^1^. Briefly, reads with similar structure were grouped into clusters of three or more alignments, with an exon boundary tolerance of 20 bp and terminal exon boundary tolerance of 60 bp. These transcript clusters were then polished and mapped back to the genome. Polished transcripts were then grouped into “loci” based on 3′ ends and collapsed to remove likely products of RNA degradation, using an internal exon boundary tolerance of 5 bp, a 3′-exon boundary tolerance of 100 bp, and a 5′-exon boundary tolerance of 5,000 bp. Because of the high prevalence of predicted single-exon transcripts, predicted transcripts were then compared to the Ensembl (v101) and NCBI RefSeq (release 106) annotations using gffcompare (v0.12.1), and only single-exon transcripts that demonstrated same-strand overlap with reference exons of protein-coding genes, or which were strongly supported (cluster size ≥ 100 alignments), were retained in the final transcript set. The set of predicted transcripts was converted to GTF format using gffread (v0.12.2) and visualized in the Integrated Genomics Viewer (v2.8.9). To visualize repetitive elements, the RepeatMasker track was downloaded from the UCSC genome annotation database for the April 2018 ARS-UCD1.2/bosTau9 assembly.

### Comparing Predicted Transcripts to Reference Annotations

Based on gffcompare class codes, predicted transcripts were classified as known isoforms of a reference gene (class code “=” when comparing to either annotation), novel isoforms of a reference gene (class codes ‘c,’ ‘k,’ ‘j,’ ‘m,’ ‘n,’ or ‘o’ when comparing to either annotation, never ‘=’), novel loci (class codes ‘i,’ ‘u,’ ‘y,’ or ‘x’ when comparing to either annotation, never ‘ =,’ ‘c,’ ‘k,’ ‘j,’ ‘m,’ ‘n,’ or ‘o’), or potential artifacts (class codes ‘e,’ ‘s,’ or ‘p’ when comparing to either annotation, but never any other class codes).

### Characterization of Predicted Transcripts

To determine the novelty of start and end sites of predicted novel isoforms, the TSS and TES of predicted novel isoforms were compared to the TSS and TES of the closest matching reference transcripts (based on gffcompare output). The usage of alternative polyadenylation sites for reference Ensembl transcripts was determined using TAPAS (Arefeen et al., 2018) with read length set to 750 bp, which was the mean read length according to the Nanoplot report. As input for TAPAS, genome-wide read depth was determined with Samtools (v1.7). The prevalence of different alternative splicing events in the final set of predicted transcripts was determined with SUPPA (v2.3), using the function generateEvents to identify local events, including skipped exons, mutually exclusive exons, retained introns, alternative 5′ or 3′ splice sites, and alternative first and last exons. Finally, the coding potential of predicted transcripts was calculated with CPPred (Tong and Liu, 2019) using the built-in human model with default parameters. To determine if predicted intergenic transcripts (gffcompare class code ‘u’) preferentially occurred near annotated genes, distance from each predicted intergenic transcript to the nearest reference gene was calculated using Bedtools closest (v2.26.0) with option “-d.” For comparison, the genomic coordinates of predicted intergenic transcripts were randomized with Bedtools shuffle (excluding regions that were already annotated as genes by Ensembl or NCBI), and these coordinates were also compared to reference genes using Bedtools closest. The distance between predicted intergenic transcripts and the closest reference genes was compared to the distance between randomized coordinates and the closest reference genes with an independent 2-group Mann–Whitney *U*-test.

### Inferring Biological Functions of Predicted Transcripts at Novel Loci

To interpret the function of predicted transcripts at novel loci, their sequences were compared against several databases. First, sequences were compared against the NT (NCBI non-redundant nucleotide, v5) database with BLASTN (v2.6.0), requiring a minimum e-value of 1e-10 for matches. Then, sequences were compared against the NR (NCBI non-redundant protein, v5) and SwissProt (downloaded from NCBI, v5) databases with Diamond BLASTX (v2.0.5.143), again setting the minimum *e*-value to 1e-10. For transcripts with SwissProt matches, the corresponding UniProt identifiers were associated with functional terms using DAVID (v6.8), including KEGG terms, GO “DIRECT” terms, and Clusters of Orthologous Groups of proteins (COG) ontology terms.

### Predicted Transcript Expression Quantification

To determine the expression of predicted transcripts, reads were directly mapped to the predicted transcriptome. Predicted transcripts were converted from GTF to FASTA format with the gffread utility (v0.12.2). Strand-corrected full-length ONT reads (output of Pychopper) were then directly mapped to the predicted transcriptome using minimap2 (v2.16r922) with options “-t 10 -ax map-ont -p 0.” Alignments with a minimum quality score of 10 were extracted with Samtools (v1.7). From these alignments, expression of predicted transcripts in transcripts per million (TPM) was determined with Nanocount (v2.3.0). For the identification of tissue-specific transcripts, samples with unclear identity were excluded. These samples included those that did not cluster with biological replicates (abomasum-F1, colon-F1, and lung-M1), tissues with unclear identity because samples did not cluster together (esophagus, skin and thyroid), and tissues with only a single replicate (duodenum-M1, hypothalamus-M1, and uterine endometrium-F1).

### Identification and Characterization of Tissue-Specific Transcripts

The tissue specificity index (TSI) (Julien et al., 2012) for each transcript was calculated as follows, such that *x*_*i*_ was the average expression (TPM) in a given tissue, and *n* was the number of tissues:

$$T\,S\,I=\frac{\max_{1\leq i\leq n}\left(x_{i}\right)}{\sum_{i=1}^{n}x_{i}}$$

Transcripts were then categorized as tissue-specific (TSI ≥ 0.8), broadly expressed (TSI < 0.5), or biased toward a group of tissues (0.5 ≤ TSI < 0.8). To interpret the biological significance of tissue-specific transcripts, those with corresponding Ensembl IDs were submitted to DAVID (v6.8) for functional enrichment analysis, considering only GO “DIRECT” terms. In each case, the top five most significant GO terms were reported (Benjamini-corrected *p*-value < 0.05). Finally, to determine whether the TSS used by tissue-specific transcripts were uniquely active in that tissue, the coordinates of TSS (±50 bp) for tissue-specific transcripts for a given tissue were extracted and compared to the TSS (±50 bp) of every other predicted transcript using Bedtools (v2.26.0) intersect, with option “-s” to only consider same-strand overlap. The TSS from tissue-specific transcripts that did not overlap any other TSS from the remaining set of predicted transcripts were considered uniquely active in that tissue.

## Results

Total RNA was extracted from 93 biological samples and used to generate cDNA libraries, which were multiplexed and sequenced on a single PromethION flow cell. Samples consisted of 32 tissues collected from two male (M1, M2) and two female (F1, F2) adult Line 1 Hereford cattle. These animals were specifically chosen for their relation to Dominette, the individual sequenced for the original cattle reference genome. Sequencing yielded 53.7 million reads, with a read length N50 of 893, average read length of 759 bases, and average quality of 8.8 (Supplementary Figure 1). After demultiplexing, 35.3 million reads passed quality thresholds (greater than Q7), and further processing yielded 30.3 million full-length strand-oriented reads which were aligned to the ARS-UCD1.2 assembly, resulting in 25.5 million unique alignments that could be used for transcript prediction (Supplementary Table 2). On average, about 270,000 reads were obtained per sample (Supplementary Table 2), and about 800,000 reads were obtained per tissue (Supplementary Table 3).

**FIGURE 1:**
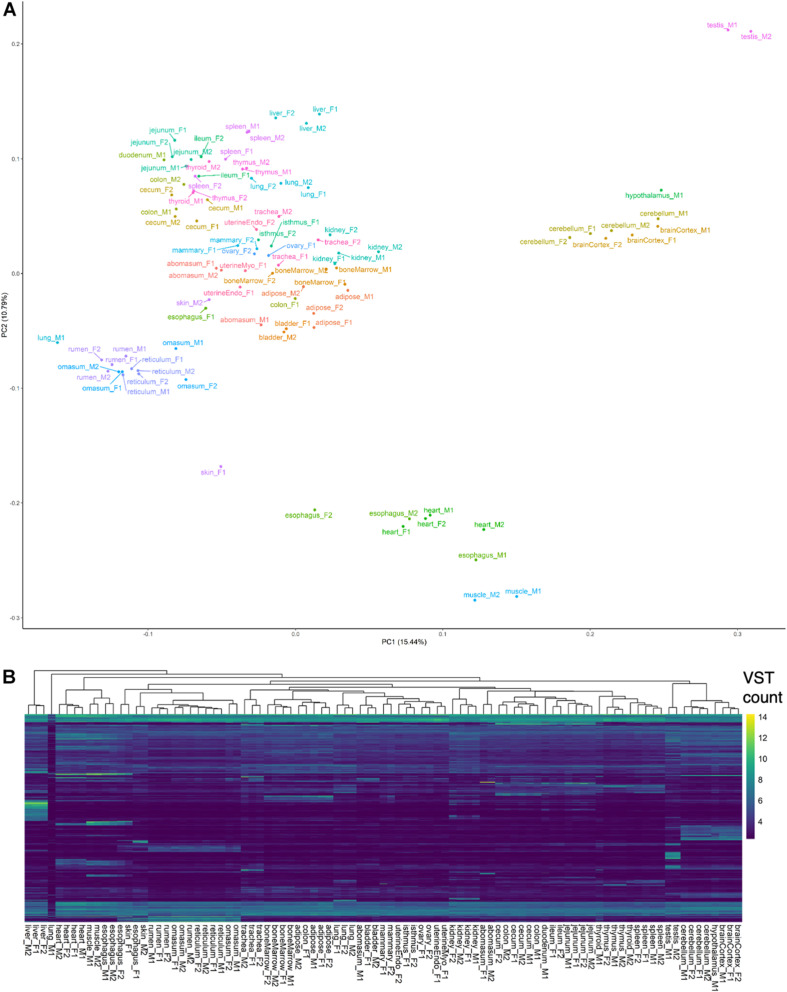
Preliminary analysis of transcriptomes. **(A)** Principal components analysis of VST-normalized gene counts. **(B)** Hierarchical clustering of samples based on top 5,000 genes with highest variance in VST counts.

A preliminary evaluation of gene expression was conducted by counting alignments attributed to genes in the Ensembl (v101) annotation (Supplementary Data 1). Principal components analysis and hierarchical clustering of normalized gene expression generally clustered samples by tissue and organ system (Figure 1), with the exception of lung-M1, which was attributed extremely few reads, abomasum-M1 and colon-F1, which did not cluster with biological replicates, and esophagus, skin, and thyroid samples, which clustered ambiguously. In particular, male esophagus samples clustered with muscle, whereas female esophagus clustered with skin and stomach samples, suggesting potential sampling error during collection of male esophagus. Samples of questionable origin, based on aberrant clustering patterns, were excluded from tissue-specific analyses, but retained in the complete dataset for predicting transcript models. Brain and testis were among the most informative tissues, based on transcriptomic complexity and number of expressed loci (Supplementary Figure 2A).

Mapped reads from all samples were pooled to predict transcript models using the Pinfish pipeline. Briefly, transcripts were predicted from clusters of three or more alignments. Predicted transcripts were then polished and collapsed to filter out likely degradation products. In total, 244,945 transcript models were predicted, consisting of 76,110 multi-exon and 168,835 single-exon transcripts. Multi-exon transcripts localized to 23,694 loci, of which 13,053 (55%) corresponded to multiple transcripts. Comparing the predicted multi-exon transcripts to Ensembl and NCBI gene annotations revealed high precision, particularly at the base and intron levels, with most reference exons and introns captured by the predicted multi-exon transcripts (Table 1).

**TABLE 1:** Sensitivity and precision estimates of predicted multi-exon transcripts compared to reference multi-exon transcripts from the Ensembl (v101) and NCBI (release 106) annotations.

	Predicted vs. Ensembl		Predicted vs. NCBI		NCBI vs. Ensembl	
			
Level	Sensitivity	Precision	Sensitivity	Precision	Sensitivity	Precision
Base	58.5	72.5	49.6	81.2	88.7	66.3
Exon	54.8	58.3	55.7	66.5	81.3	73.2
Intron	60.1	80.7	57.3	86.5	90.0	79.4
Transcript	29.3	12.9	24.6	20.2	48.8	26.7
Locus	52.6	47.9	62.7	56.8	75.6	76.9
Missed exons	53,069/171,341 (31.0%)		65,592/207,468 (31.6%)		8,891/222,022 (4.0%)	
Novel exons	15,130/193,597 (7.8%)		8,366/203,236 (4.1%)		27,725/257,826 (10.8%)	
Missed introns	48,381/151,779 (31.9%)		55,075/177,905 (31.0%)		3,643/195,870 (1.9%)	
Novel introns	4,528/112,911 (4.0%)		2,447/117,961 (2.1%)		12,338/222,064 (5.6%)	

*Comparison excludes reference loci without predicted transcripts and predicted transcripts at novel loci.*

Compared to multi-exon transcripts, single-exon transcripts were supported by fewer reads (*p* < 2.2e-16; one-sided *Z*-test) (Supplementary Figure 3A), and tended to not directly overlap annotated exons, instead occurring predominantly within reference introns (Supplementary Figure 3B). Consequently, only single-exon transcripts that corresponded to annotated protein-coding genes, or those which were supported by more than 100 alignments (i.e., the top 1% most strongly supported single-exon transcripts) (Supplementary Figure 4), were retained in the final transcript set, which comprised 99,044 predicted transcripts (22,934 single-exon and 76,110 multi-exon transcripts) belonging to 31,824 genomic loci. Although only a small percentage of the retained single-exon transcripts were predicted to be coding (5%), the expression patterns of single-exon transcripts clearly distinguished brain tissues from the others (Supplementary Figure 5), suggesting these transcripts are biologically relevant. Expression of non-coding transcripts also distinguished brain, as well as testis, from other tissues (Supplementary Figure 6). Overall, transcript predictions accounted for 72% (15,716/21,861) of protein-coding genes in the Ensembl annotation and 78% (16,487/21,039) of protein-coding genes in the NCBI annotation.

**FIGURE 2:**
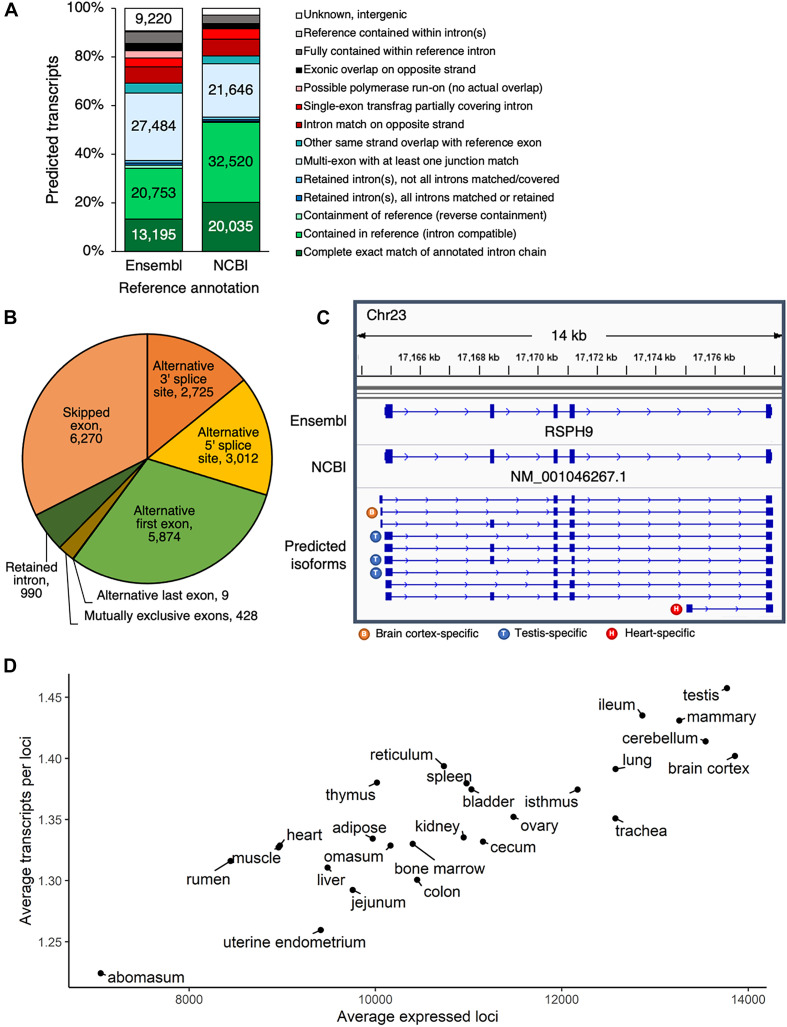
Predicted transcripts capture transcriptome complexity. **(A)** Comparison of predicted isoforms to Ensembl and NCBI gene annotations. **(B)** Frequency of alternative splicing events in predicted multi-exon transcript isoforms. **(C)** Predicted isoforms at the RSPH9 locus, which is thought to code for a component of motile cilia and flagella. In humans, multiple splicing is known to produce transcript variants, but only one transcript had been annotated in cattle, according to both the Ensembl and NCBI annotations. **(D)** Based on the predicted transcript set, number of expressed loci and ratio of expressed transcripts per loci, averaged per tissue.

Comparing the predicted transcript set to either the Ensembl or the NCBI annotations (Supplementary Data 2, 3) revealed that most predicted transcripts either exactly matched a reference transcript exon-by-exon, or demonstrated some same strand overlap with reference exons (Figure 2A). In all, 21% of predicted transcripts exactly matched a reference transcript from either Ensembl or NCBI, 61% were considered novel isoforms of reference genes based on same strand overlap of reference exon(s), 6% did not correspond to a reference gene and were considered novel loci, and 12% were classified as potential artifacts, possibly due to mapping error, pre-mRNA fragments, or polymerase run-on.

Considering the largest class of predicted transcripts were novel isoforms of known genes, we then sought to quantify the extent to which variation in transcription start sites, end sites, alternative splicing, and alternative polyadenylation sites contributed to transcriptome complexity. Transcript degradation, especially at the 5′ end, is certainly a concern in long-read transcriptomics, although the 3′ ends are considered to be more reliable. The Pinfish pipeline used to predict transcripts tries to take this limitation into account by collapsing transcripts with similar exon structure and variable 5′ ends, within a 5,000 bp 5′ exon boundary tolerance. Considering all 5′ ends of predicted transcripts (±100 bp), we found that 28% overlapped 5′ ends of Ensembl or RefSeq transcripts (±100 bp), and 45% overlapped TSS (±100 bp) identified by the 5′-complete sequencing technique RAMPAGE (Goszczynski et al., 2020). Even when predicted 5′ ends did not directly coincide with Ensembl, RefSeq or RAMPAGE annotations (Supplementary Data 4), they still preferentially occurred in the vicinity of RAMPAGE TSS (39% of these 5′ ends occurred within 1kb of RAMPAGE TSS) and were not biased downstream of RAMPAGE TSS (Supplementary Figure 7), which would have been characteristic of degradation. Most novel isoforms began within 2 kb of the reference transcription start site (51%, 28,289 transcripts) and terminated within 2 kb of the reference transcription end site (58%, 31,913 transcripts) (Supplementary Figure 8). Additional variation was present at TES, as alternative polyadenylation sites were detected for 30% of reference Ensembl transcripts (5,821/19,613 transcripts) (Supplementary Figure 9).

The main source of transcriptional variation resulted from alternative splicing (Figure 2B). Alternative first exons were common in predicted multi-exon transcripts, reflecting the use of alternative promoters in different regulatory contexts. This phenomenon was clearly reflected at the *RSPH9* locus, which encodes a component of motile flagella and is associated with multiple transcript variants from alternative splicing in humans, although only a single isoform had been annotated in cattle (Figure 2C). Besides the alternative splicing evident at this locus, three different transcription start sites were utilized, resulting in ten isoforms, several of which demonstrated tissue-specific expression patterns (Supplementary Figure 10). In a given tissue sample, 10,844 ± 2,010 (S.D.) loci were expressed with 1.35 ± 0.06 (S.D.) predicted isoforms expressed per locus. Testis was the most informative tissue, with the most expressed loci and highest ratio of expressed transcripts per gene, whereas abomasum demonstrated the lowest transcriptomic complexity (Figure 2D).

Given the large number of sampled tissues, tissue-specific isoforms could be identified from this dataset with high resolution. Tissue-specific transcripts are fundamental to understanding the basis of biological differences between tissues, and can serve as useful biomarkers (Stutterheim et al., 2008; Prensner et al., 2013), as they are often implicated in tissue-specific functions, development, and disease (Leucci et al., 2016). To identify tissue-specific isoforms, the tissue-specificity index (TSI) was calculated from the average expression of predicted transcripts (transcripts per million; TPM) in each tissue with at least two high-confidence biological replicates (adipose, bladder, bone marrow, brain cortex, cecum, cerebellum, colon, heart, ileum, isthmus, jejunum, kidney, liver, lung, mammary gland, muscle, omasum, ovary, reticulum, rumen, spleen, testis, thymus, trachea, and uterine endometrium) (Supplementary Data 5). For a given transcript, the TSI varies between 0 (uniformly expressed across all tissues) and 1 (uniquely expressed in a single tissue). Transcripts that were only expressed in a single sample were excluded from the tissue-specificity analysis.

**FIGURE 3:**
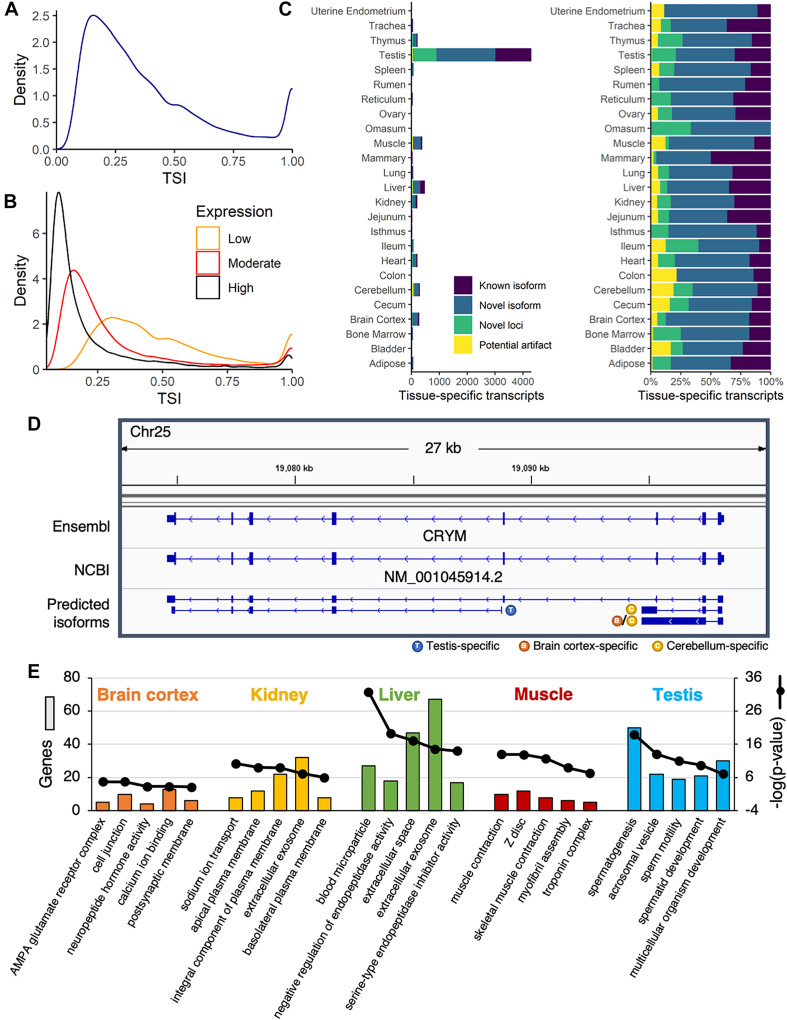
Identification of tissue-specific isoforms. **(A)** Density plot of the tissue-specificity index (TSI) identified for each predicted transcript, based on average transcripts per million (TPM) in each tissue. **(B)** Density plot of TSI for predicted transcripts with low (average TPM < 1), moderate (1 ≤ average TPM < 10), or high expression (average TPM ≥ 10). **(C)** Number of tissue-specific transcripts (TSI ≥ 0.8) attributed to each tissue, categorized as known or novel isoforms, novel loci, or potential artifacts. **(D)** The annotated transcript at the CRYM locus was expressed across a range of tissues, whereas novel isoforms were either testis- or brain-specific. **(E)** Functional enrichment of genes corresponding to tissue-specific isoforms in brain cortex, kidney, liver, muscle, and testis. Top five most significant gene ontology terms reported (Benjamini corrected *p*-value < 0.05).

Overall, the TSI demonstrated a bimodal distribution, with most transcripts either broadly (TSI closer to zero) or specifically (TSI closer to 1) expressed (Figure 3A). This pattern was observed for both single- and multi-exon transcripts (Supplementary Figure 11A). The TSI was closely linked to the average expression across samples, with highly expressed transcripts (average TPM ≥ 10) more often generally expressed across many tissues, whereas moderately- (1 ≤ average TPM < 10) and lowly expressed transcripts (average TPM < 1) tended to be more tissue-specific (Figure 3B). Overall, 48,867 transcripts (74%) were widely expressed (TSI < 0.5), 7,066 transcripts (11%) were highly tissue-specific (TSI ≥ 0.8), and 10,203 transcripts (15%) demonstrated expression in a small subset of tissues (0.5 ≤ TSI < 0.8). Interestingly, compared with multi-exon transcripts, single-exon transcripts were more likely to be brain-specific (Supplementary Figure 11B), and were generally predicted to be non-coding (95%), which is consistent with the central role of non-coding RNA in the brain (Guennewig and Cooper, 2014). Transcripts with intermediate TSI scores likely includes isoforms specific to higher-order structures from which multiple tissues were sampled (e.g., brain, pre-stomach, gastrointestinal tract), or tissues of similar embryonic origin (e.g., ectodermal, mesodermal, endodermal) as has been observed by previous transcriptomic studies in the pig (Perez-Montarelo et al., 2012).

An overwhelming proportion of tissue-specific transcripts (61%) were attributed to testis, and most of these were either novel isoforms (49%) or novel loci (20%) (Figure 3C). More than 80% of the transcription start sites used by testis-specific isoforms were only active in testis (Supplementary Figure 12), suggesting pervasive use of alternative promoters in this tissue. This alternative promoter usage was evident at the *CRYM* locus, with a novel testis-specific isoform beginning at the third annotated exon (Figure 3D). The remaining novel *CRYM* isoforms were brain-specific, whereas the sole annotated transcript variant was broadly expressed across tissues. This locus illustrated a broader pattern: novel isoforms of annotated genes were expressed in fewer tissues and at lower levels than previously annotated isoforms (*p* < 2.2e-16; Welch two sample *t*-test) (Supplementary Figure 13), suggesting that the reference genome annotations failed to capture rare isoforms with potentially significant biological functions. Indeed, genes with tissue-specific isoforms were strongly biased toward tissue-specific functions (Figure 3E).

**FIGURE 4:**
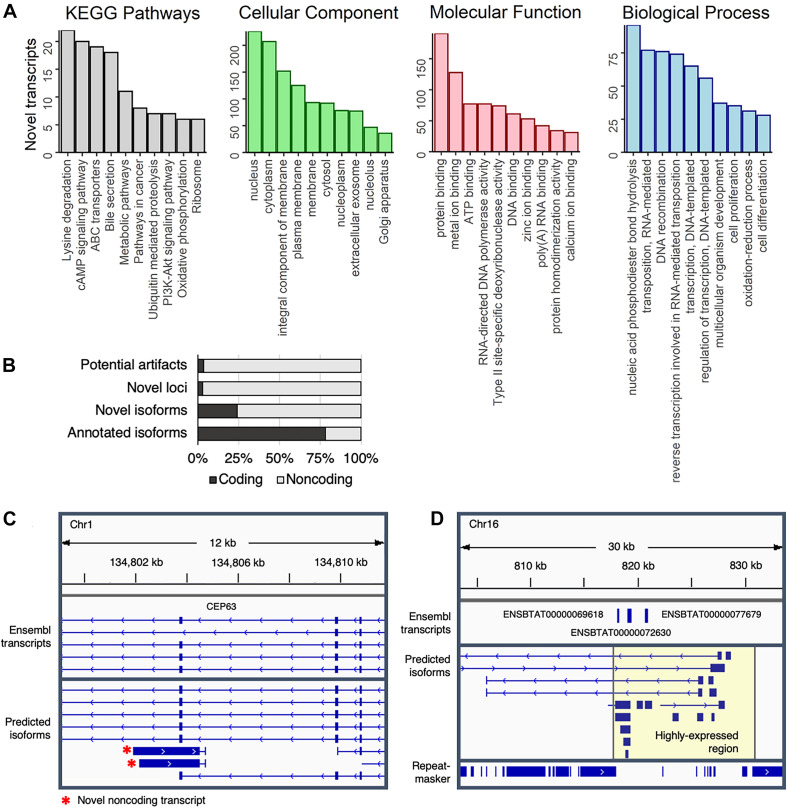
Characterization of predicted transcripts at novel loci. **(A)** The top ten represented KEGG pathways and GO terms (separated into Cellular Component, Molecular Function, and Biological Process terms) represented in transcripts at novel loci that corresponded to a UniProt identifier. **(B)** Coding potential of predicted transcripts. **(C)** Novel non-coding antisense transcript at the CEP63 locus. **(D)** Highly expressed section of chromosome 16. RepeatMasker track shows repetitive elements, which were depleted in the highly expressed region (highlighted in yellow).

To gain some insight into the potential biological functions of isoforms at novel loci, transcript sequences were compared against several BLAST databases (Supplementary Data 6). Strong matches (*E*-value < 1e-10) were identified for 93% (5,944/6,370) of transcripts at novel loci when comparing against the NT database (NCBI non-redundant nucleotide sequences), 42% (2,678/6,370) against the NR database (NCBI non-redundant protein sequences), and 12% (794/6,370) against the SwissProt database (curated protein sequences). Based on gene ontology (GO) terms and KEGG pathways associated with SwissProt identifiers, transcripts at novel loci are involved in a variety of biological functions, such as lysine degradation, cAMP signaling, and phosphodiester bond hydrolysis (Figure 4A). Of note, two of the top ten most common biological process GO terms were related to RNA-mediated transposition, indicating that some novel transcripts could correspond to transposons that have not been completely silenced.

The genomic distribution of novel loci was biased toward contigs; whereas only 0.4% of all predicted transcripts (342/99,044) localized to contigs, 7.7% of transcripts at novel intergenic sites (126/1,628) were on contigs. Nevertheless, novel intergenic transcripts preferentially occurred closer to annotated genes (on average 60 kb away from an Ensembl transcript) than would be expected by random chance (on average 140kb away from an Ensembl transcript) (*p* < 2.2e-16; Independent two-group Mann–Whitney *U*-test) (Supplementary Figure 14). Transcripts at novel loci tended to be shorter than those of annotated genes with fewer exons (Supplementary Figure 15), despite the exclusion of most intergenic single-exon predicted transcripts.

Nearly all predicted transcripts at novel loci appeared to be non-coding (Figure 4B), which could partially explain the lower number of matches in protein-based databases (NR and SwissProt) as compared to the nucleotide-based database (NT). For instance, transcription of the anti-sense strand at the *CEP63* locus – a centrosomal protein crucial for division of brain cells – produces short (∼2 kb long) non-coding transcripts (Figure 4C) that are expressed in a mutually exclusive pattern with the main *CEP63* isoform (Supplementary Figure 16), potentially suggesting that *CEP63* expression is regulated by a previously unannotated antisense non-coding RNA.

Of note, more than 1.5 million reads (6.5% of the entire dataset), were aligned to a single 15 kb region on chromosome 16 (Figure 4D). Surprisingly, this region contained no RefSeq transcripts, although the Ensembl annotation included three single-exon transcripts that were predicted to code for NADH hydrogenase and ATP synthase subunits. Considering this region was strongly expressed across all samples (Supplementary Figure 17), these transcripts likely serve fundamental biological roles that remain to be established. Additionally, because gene expression is generally normalized based only on reads that align to the exome, the inclusion of these loci in future annotations could improve estimates of gene expression in transcriptomic-based studies.

## Discussion

Although long-read sequencing has been extensively implemented for the study of transcription dynamics, resulting datasets have generally either been limited by sample size or sequencing depth. To address this limitation, here we demonstrate that by coupling ONT sequencing with large-scale multiplexing, we were able to profile the full-length transcriptomes of 32 adult bovine tissues from a single ONT flow cell. Of the nearly 100,000 predicted transcripts, over 60% were novel isoforms of reference genes, indicating that the complexity of the bovine transcriptome is comparable to what has been described in humans. Moreover, this high percentage of novel isoforms is consistent with other studies that have used long-read sequencing to improve annotations in pigs (80% of identified transcripts were novel), rabbits (66%), and cattle (60%) (Chen et al., 2017; Beiki et al., 2019; Rosen et al., 2020). Compared to previous efforts to annotate full-length bovine transcripts (Rosen et al., 2020), this study leveraged a single ONT flow cell to interrogate more tissues (32 versus 23) from multiple individuals (four replicates versus one) at a greater sequencing depth (25 million versus 553,798 reads). In terms of cost, speed, and throughput, these comparisons highlight the power of this method for transcriptome annotation.

Overall, our transcript predictions substantially increased the ratio of isoform variants per reference bovine gene from 1.59 to 3.57 (74,312 transcripts at 20,811 reference Ensembl loci), which is consistent with the ratio observed in humans (3.78 transcripts per reference Ensembl locus) (Supplementary Figure 18). Although not all of the 5′ ends of predicted transcripts directly overlapped Ensembl, RefSeq, or RAMPAGE TSS, the corresponding transcripts (Supplementary Data 4) were not disregarded. Just as this study cannot provide a comprehensive catalog of full-length bovine transcripts, analysis of RAMPAGE data may have missed credible TSS. Further efforts to annotate regulatory elements in bovine tissues (i.e., by profiling chromatin accessibility and histone modifications) should help to further refine the 5′ ends of transcript models; however, these data are not yet available for all tissues.

Notably, this study only profiled samples from a single breed – Hereford – which was specifically chosen because it is also the basis for the current bovine genome assembly. Consequently, these data cannot account for the substantial phenotypic and genetic variation observed between different breeds and subspecies of cattle (Weigel et al., 2017). For instance, taurine breeds are known to have higher fertility than indicine breeds, whereas indicine breeds demonstrate higher resistance to disease and parasites and thrive in hotter climates. Although a recent study reported identification of haplotype-specific transcripts by PacBio sequencing, the dataset was limited to seven tissues from a *Bos Taurus* hybrid fetus (Low et al., 2020). Moving forward, it will be of considerable scientific and economic interest to continue investigating breed-specific transcriptomes, with the goal of better understanding the biological mechanisms that underpin phenotypic differences between animals.

Although this study interrogated over 30 adult tissues, the resulting annotation is still far from exhaustive. Transcription was only detected at about 60% of reference loci; the remainder may not have been expressed in the sampled tissues, or may have been expressed at such a low level that expression was not detected due to lower sequencing depth per sample. On average, we found each tissue expressed about 10,000 loci, although some tissues – specifically brain and testis – demonstrated substantially more complex transcriptomes. On the other hand, nearly 30% of all reads attributed to abomasum samples originated from *LYZ2* (Supplementary Data 7), part of the lysozyme *c* family of digestive proteins that play an important role in ruminant digestion (Irwin, 2015). Such highly abundant transcripts can be problematic for transcriptomic studies, as they make it harder to detect rare transcripts with potential biological significance. This problem is intensified for long-read sequencing methods, which generally have lower throughput; however, it is possible to specifically target such transcripts by hybridization, for example by the CRISPR-Cas9 based method DASH (depletion of abundant sequences by hybridization) (Gu et al., 2016) which was recently employed to deplete hemoglobin transcripts prior to ONT sequencing of polar bear blood (Byrne et al., 2019). To gain a more complete picture of transcription in bovine abomasum or blood – the latter of which was not profiled in this study – it will likely be necessary to deplete abundant transcripts, such as lysozyme and hemoglobin, in order to detect rarer isoforms.

Another potential limitation of our approach is that it was based on cDNA, the generation of which is inherently limited by the capacity of reverse transcriptase to amplify long transcripts. As a result, it was difficult to capture full-length transcripts for some of the longest genes, such as titin (*TTN*), which also tended to produce fragmented Iso-seq reads (Rosen et al., 2020). In addition to fragment length limitations, PCR amplification can also introduce substantial GC-content bias into libraries (Mamanova et al., 2010), altering transcript abundance and library complexity. Furthermore, by using oligo-dT primers for cDNA generation, as opposed to random primers, our transcript predictions are likely biased against RNAs that are generally not polyadenylated (e.g., non-coding RNAs). Single-molecule sequencing platforms, such as the MinION (Garalde et al., 2018), avoid these PCR biases altogether by reading native RNA nucleotides directly as they pass through a nanoscale sensor. Moreover, direct RNA sequencing can identify post-transcriptional events like ribonucleotide modifications, which are increasingly recognized as key regulators of several biological processes (Jantsch et al., 2018).

Nevertheless, native RNA long-read sequencing is somewhat limited by throughput and transcript truncation. A single MinION flow cell produces only about half a million aligned reads (Soneson et al., 2019), as compared to the 30 million aligned reads generated by this study from a single PromethION flow cell. In addition, a significant portion of native RNA reads are truncated during Nanopore direct RNA sequencing, especially the last 10–15 nucleotides at the 5′ end (Soneson et al., 2019; Workman et al., 2019). In theory, this issue could be resolved by filtering out ONT reads that do not begin within defined promoters, which were recently experimentally determined in cattle (Goszczynski et al., 2020), but this approach would undoubtedly reduce the quantitative nature of the data.

These limitations notwithstanding, as long-read sequencing technologies continue to improve, both native RNA and single-cell ONT strategies are likely to become increasingly accurate, informative and practical, providing unprecedented insight into transcriptome complexity and cell-to-cell heterogeneity (Lebrigand et al., 2020). In fact, recent efforts to computationally correct sequencing errors in ONT data are capable of reducing the error rate from 14% (Workman et al., 2019) to about 1% (Sahlin et al., 2020), such that it should be possible for future studies to use ONT sequencing for reference-free *de novo* transcriptome analysis.

As it stands, we have demonstrated the potential for multiplexing paired with ONT sequencing as a powerful and accessible technique for isoform identification and expression profiling. Nevertheless, to comprehensively capture the transcriptomic complexity of the bovine genome, future studies will need to continue to characterize transcript isoforms in a broader range of tissues and cell types, representing different developmental stages, disease states, and physiological conditions. The ability to identify full-length transcripts from nearly one hundred samples using a single ONT flow cell makes the task of exhaustively annotating a mammalian transcriptome significantly more feasible.

## Data Availability Statement

The datasets presented in this study can be found in online repositories. The names of the repository/repositories and accession number(s) can be found below: https://www.ncbi.nlm.nih.gov/geo/, GSE160028.

## Ethics Statement

The animal study was reviewed and approved by the University of California, Davis Institutional Animal Care and Use Committee.

## Author Contributions

PR designed the study. JM and HZ contributed to the experimental design. AI-T performed the RNA extractions and generated libraries for sequencing. MH and DG performed the bioinformatics analyses. MH and PR wrote the manuscript. All the authors have read and approved the manuscript.

## Conflict of Interest

The authors declare that the research was conducted in the absence of any commercial or financial relationships that could be construed as a potential conflict of interest.

## Footnotes

1 github.com/nanoporetech/pinfish
