# Supplementary material for: Large-Scale Multiplexing Permits Full-Length Transcriptome Annotation of 32 Bovine Tissues From a Single Nanopore Flow Cell
Source: Front Genet. 2021 May 20;12:664260. doi: 10.3389/fgene.2021.664260 (PMC8173071; doi:10.3389/fgene.2021.664260)
Supplement: Supplementary file 8 [file Data_Sheet_8.pdf]

Supplementary Figures, Tables, and Data

Large-scale multiplexing permits full-length transcriptome annotation of 32 bovine tissues from a single Nanopore flow cell

Halstead et al.

Supplementary Data 1. Gene-level raw expression counts (Ensembl v101 annotation) prior to transcript predictions.

Supplementary Data 2. Predicted bovine transcripts in GTF format, as compared to the Ensembl annotation (v101).

Supplementary Data 3. Predicted bovine transcripts in GTF format, as compared to the NCBI RefSeq annotation (release 106).

Supplementary Data 4. List of predicted transcripts with 5' ends that did not correspond to Ensembl, RefSeq, or RAMPAGE annotations.

Supplementary Data 5. Transcript-level normalized counts (transcripts per million; TPM) for predicted transcripts.

Supplementary Data 6. Novel transcript characterization, including transcript length, coding potential, BLAST hits from (NT, NR, and SwissProt databases), and associated functions (GO terms, KEGG pathways, and COG terms).

Supplementary Data 7. Top three most highly expressed genes per sample.

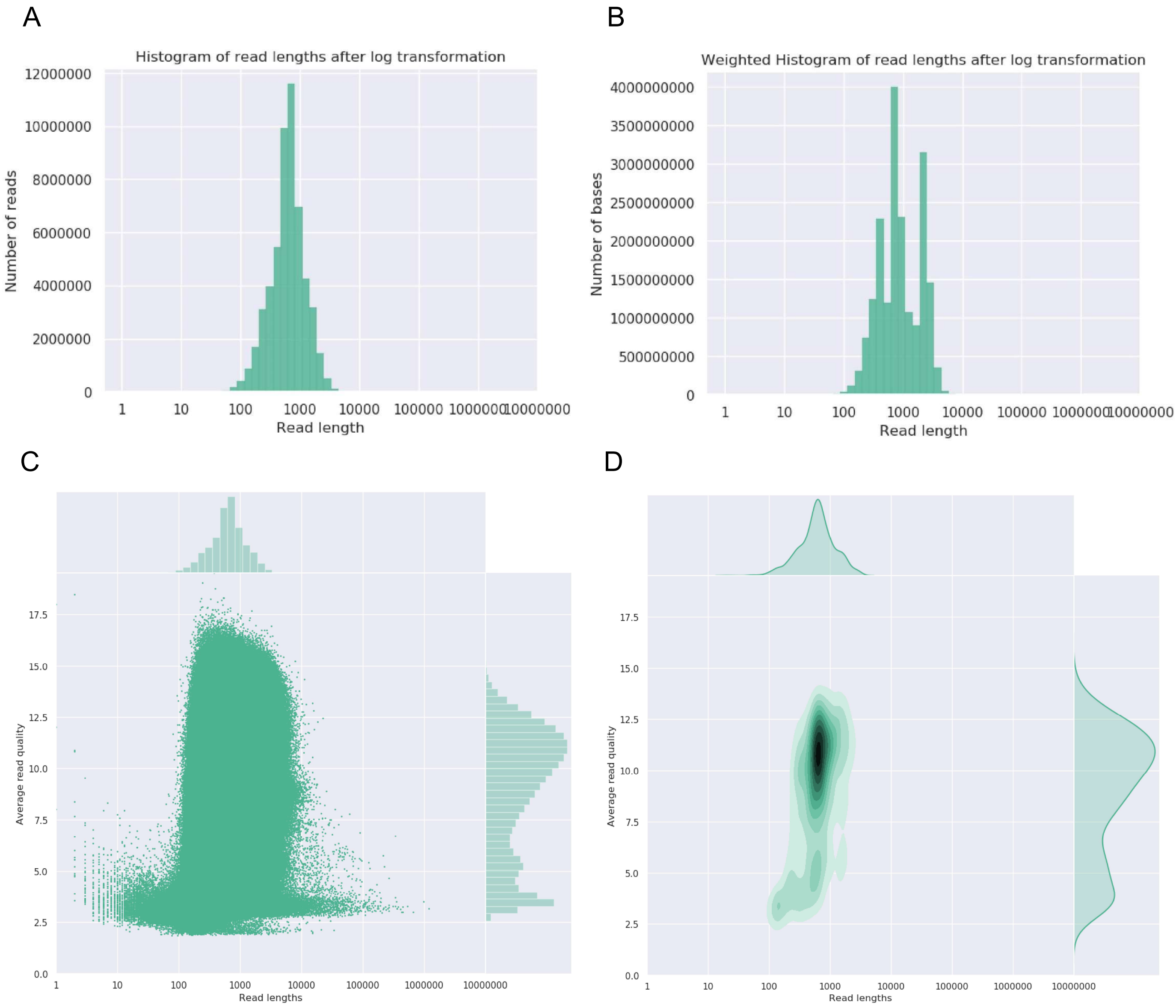

Supplementary Figure 1. Quality control of non-demultiplexed raw ONT sequencing reads. (A) Histogram of read lengths after log transformation. (B) Weighted histogram of read lengths after log transformation. (C) Bivariate plot comparing read lengths after log transformation with average Phred quality score. (D) Bivariate density plot comparing read lengths after log transformation with average Phred quality score.

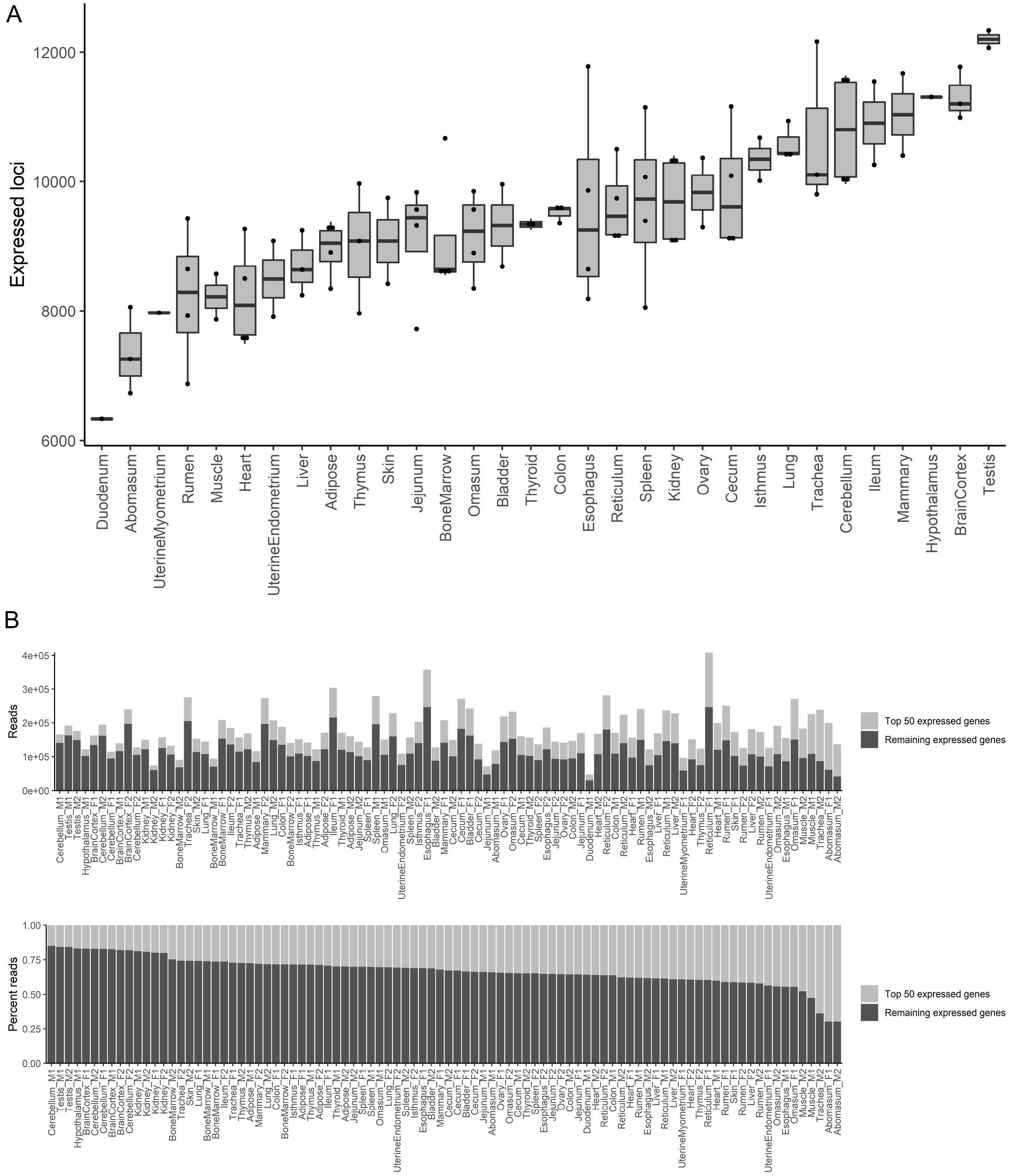

Supplementary Figure 2. Tissue transcriptome complexity. (A) Number of Ensembl loci (genes) that were attributed at least one full-length uniquely aligned reads in each sample, summarized by tissue, and sorted by average number of expressed loci. (B) For each sample, proportion of full-length uniquely aligned reads attributed to the top 50 most highly expressed loci.

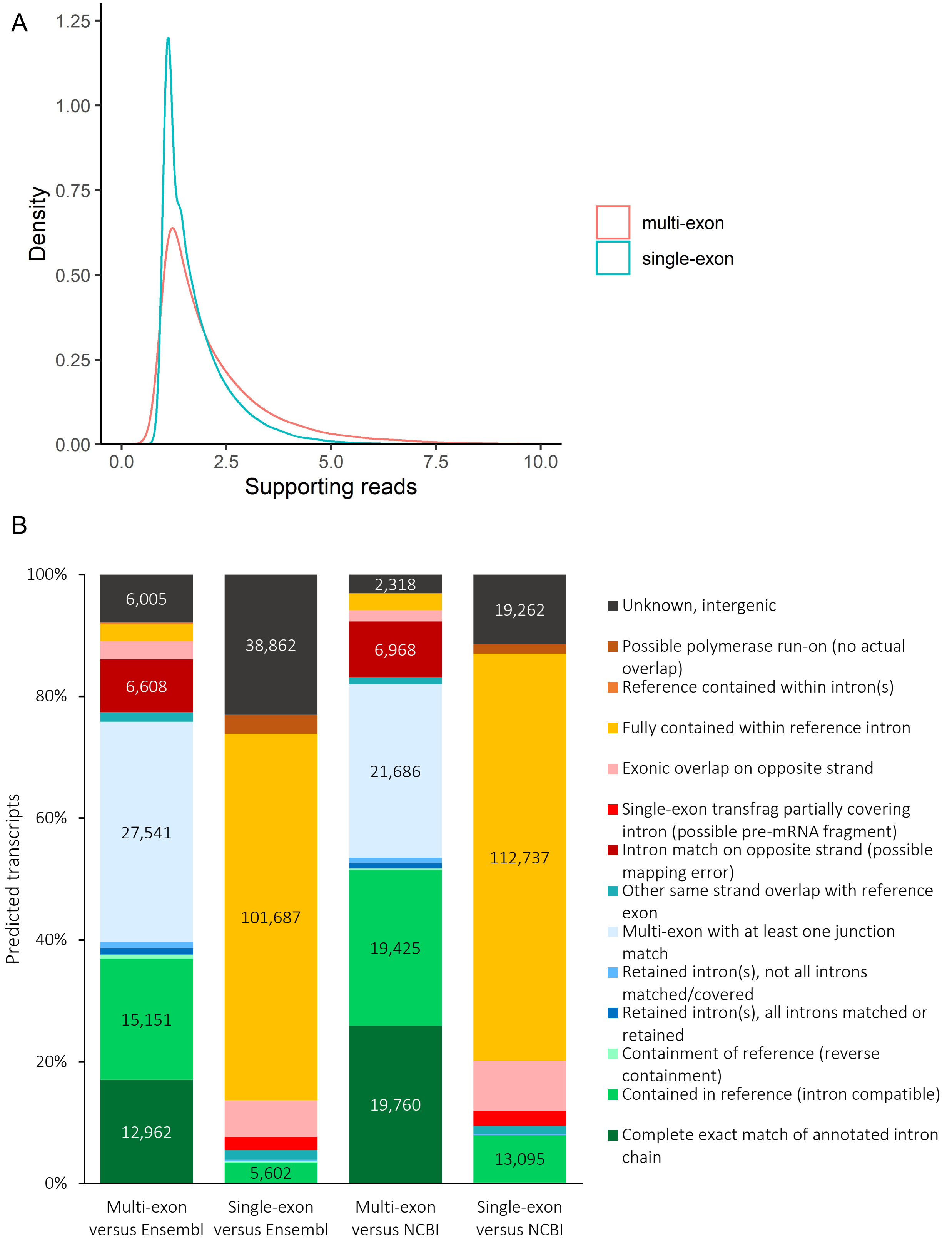

Supplementary Figure 3. Predicted single- and multi-exon transcripts. (A) Density plots showing number of reads supporting single- or multi-exon transcripts. (B) Localization of all predicted transcripts relative to the Ensembl (v101) or NCBI (release 106) gene annotations.

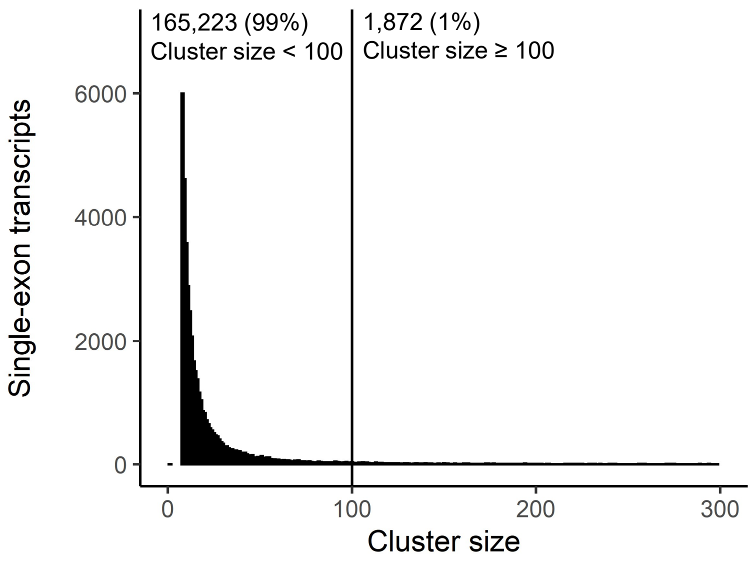

Supplementary Figure 4. Distribution of cluster size (i.e., number of supporting read alignments) for all 168,835 predicted single-exon transcripts. Only single-exon transcripts with a cluster size ≥ 100 (the top 1% of supported single-exon transcripts) were retained in the final set of predicted transcripts.

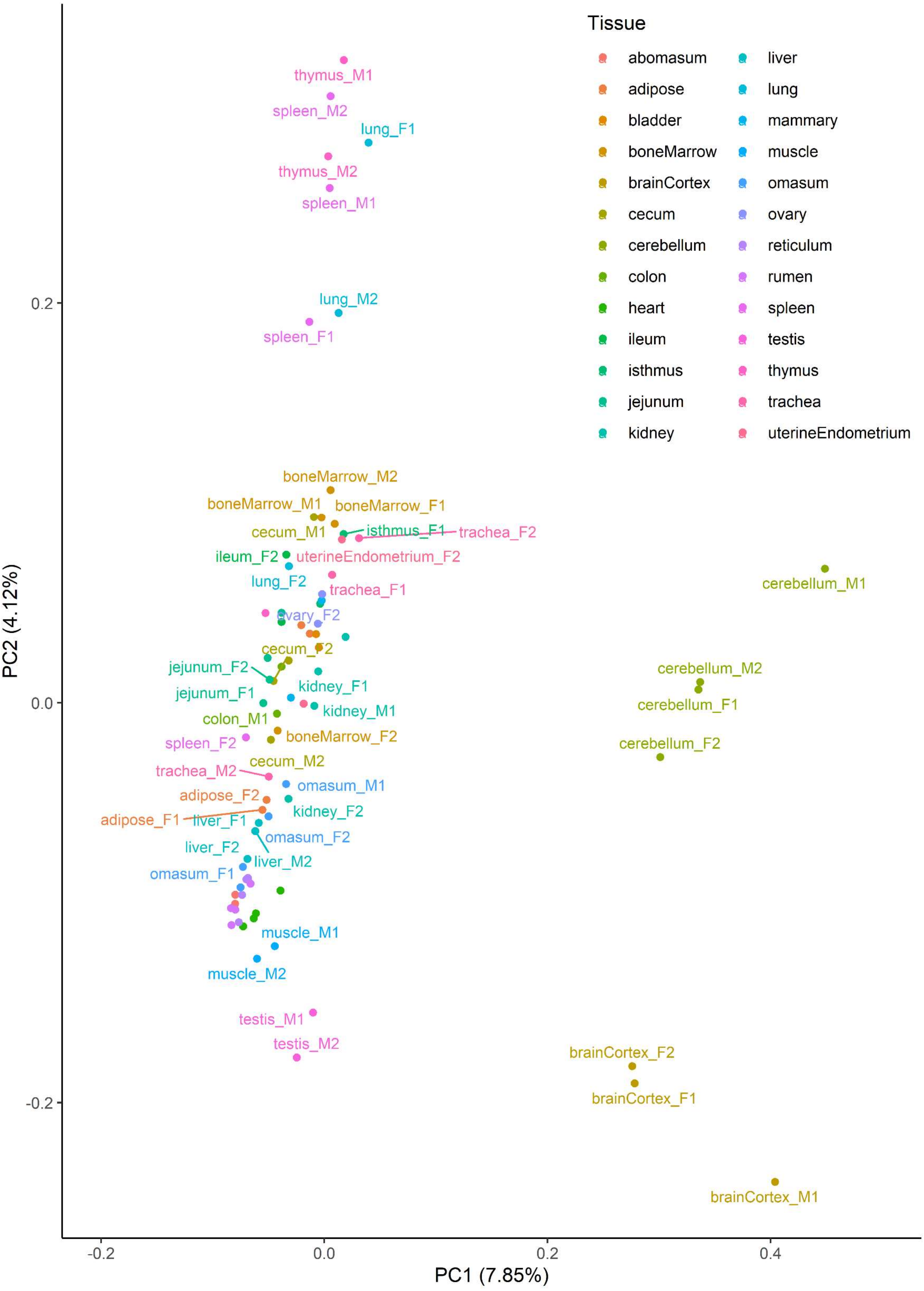

Supplementary Figure 5. PCA plot of samples, based on normalized expression (TPM) of single-exon transcripts.

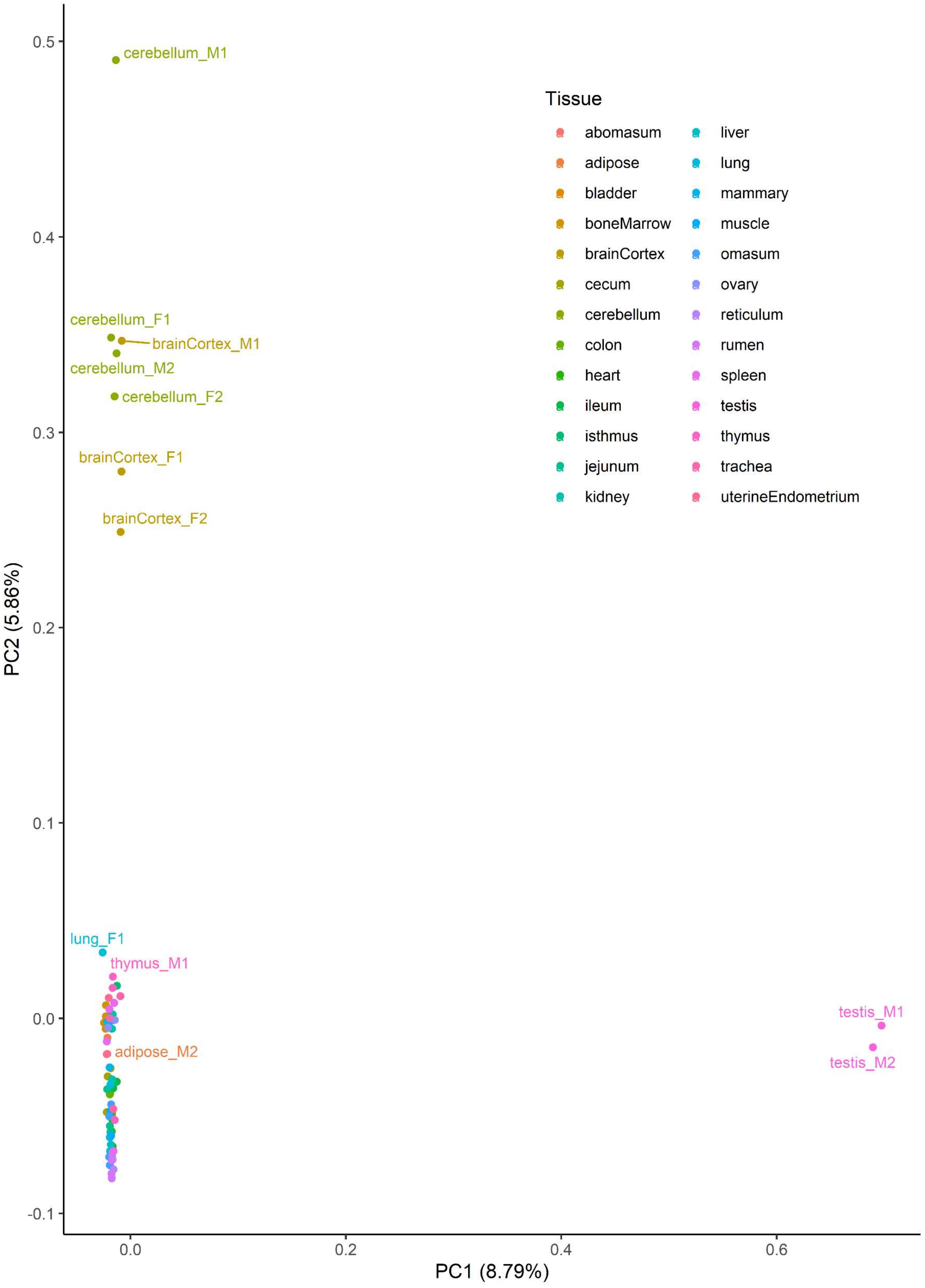

Supplementary Figure 6. PCA plot of samples, based on normalized expression (TPM) of non-coding transcripts.

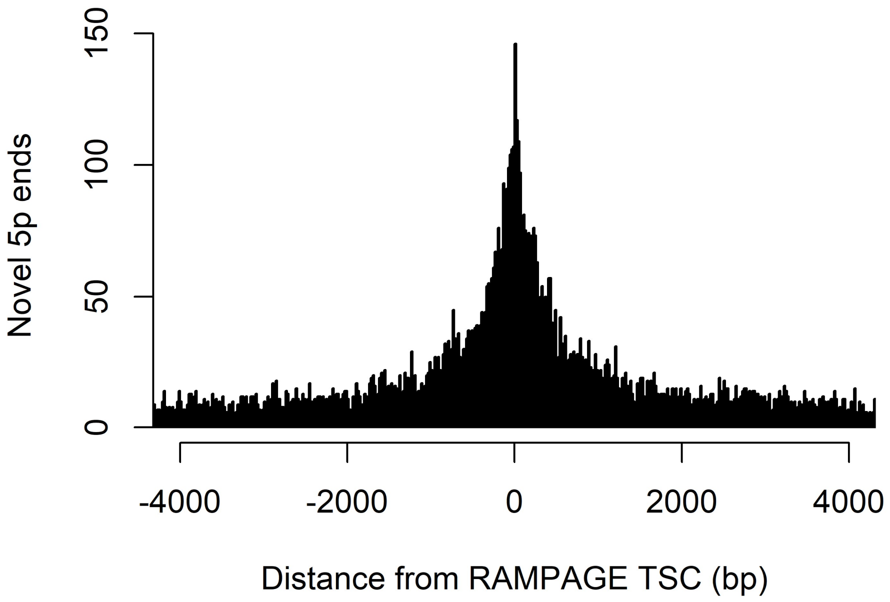

Supplementary Figure 7. Distance between novel 5' ends of predicted transcripts relative to transcription start site clusters (TSC) predicted from RAMPAGE data. Novel 5' ends (± 100bp) were those that did not directly intersect with annotated 5' ends (Ensembl and Refseq, ± 100bp) or RAMPAGE TSCs (± 100bp). Positive values indicate novel 5' ends that were downstream of RAMPAGE TSC, and negative values indicate novel 5' ends were upstream of TSC.

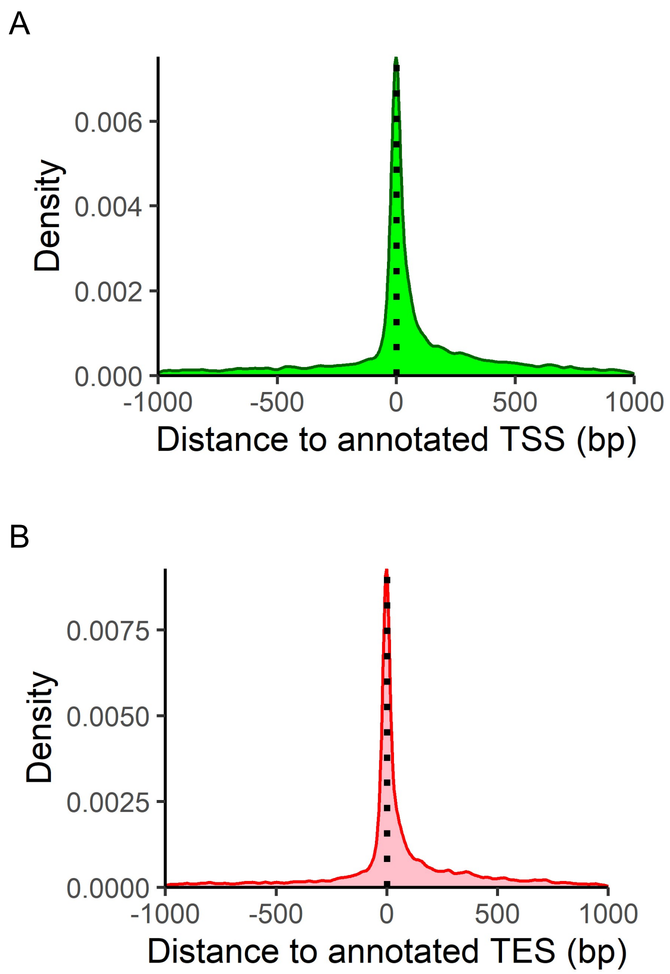

Supplementary Figure 8. Localization of transcription start (TSS) and end sites (TES) of novel isoforms relative to reference (A) TSS and (B) TES from the Ensembl annotation (v101).

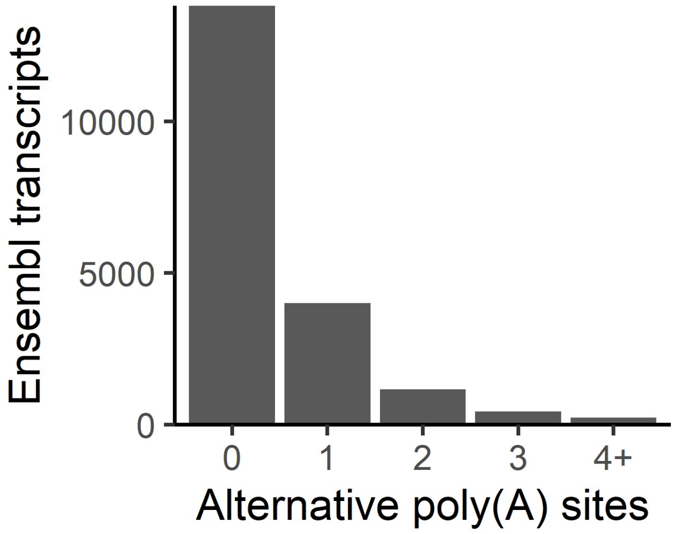

Supplementary Figure 9. Frequency of alternative poly(A) sites detected from ONT sequencing data for Ensembl (v101) reference transcripts.

A

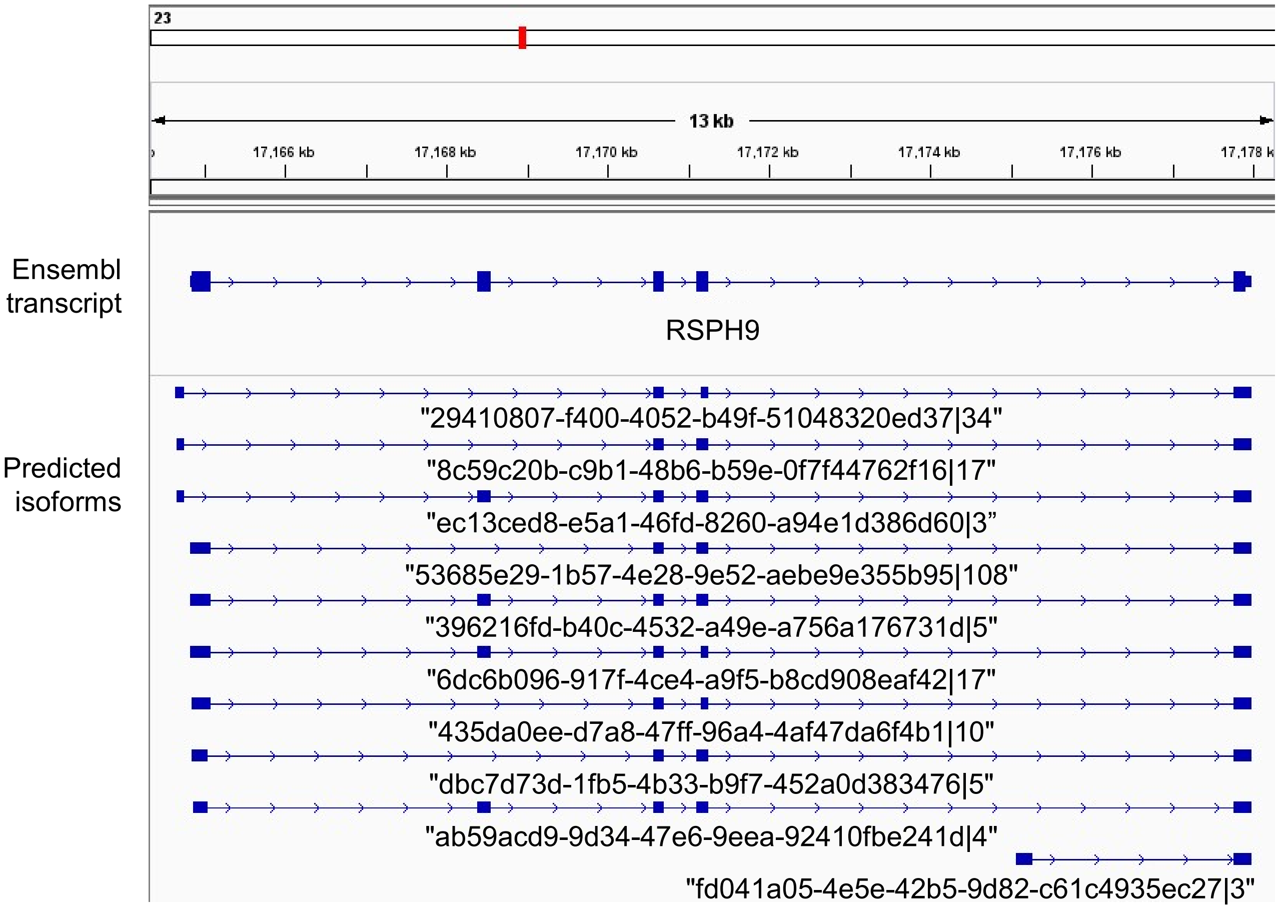

B

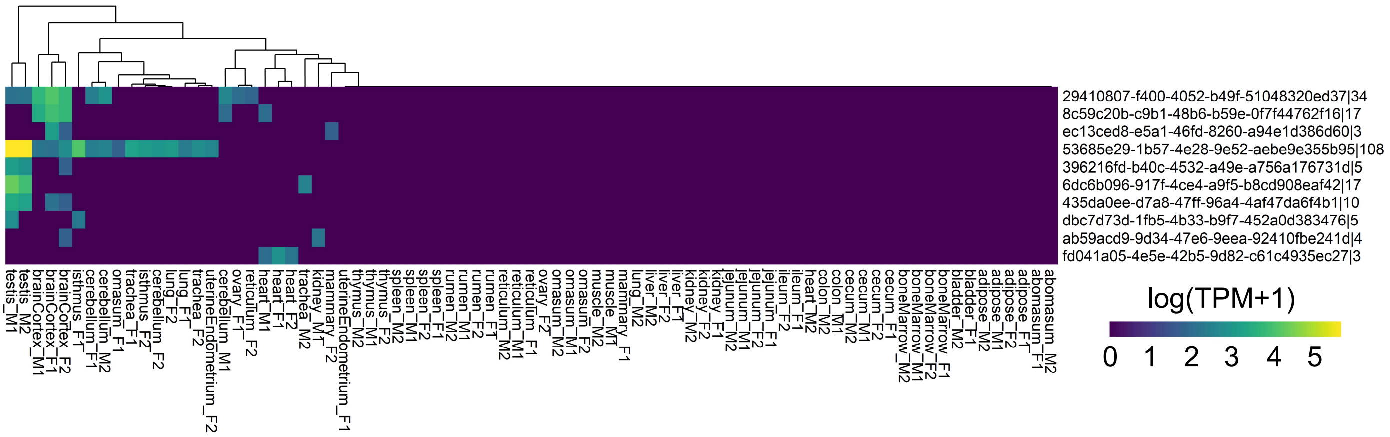

Supplementary Figure 10. Transcript variation and expression of the RSPH9 locus. (A) Detailed view of predicted *RSPH9* variants. (B) Expression profiles of predicted RSPH9 transcript variants.

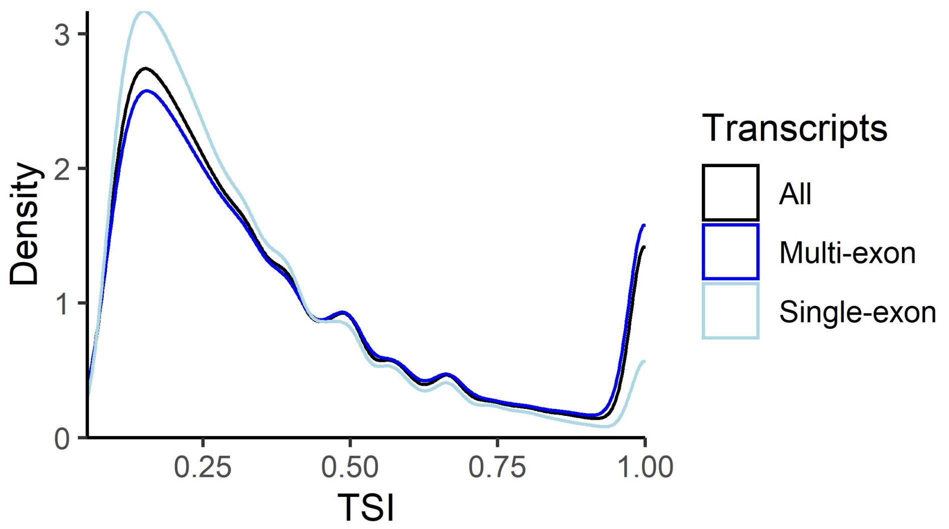

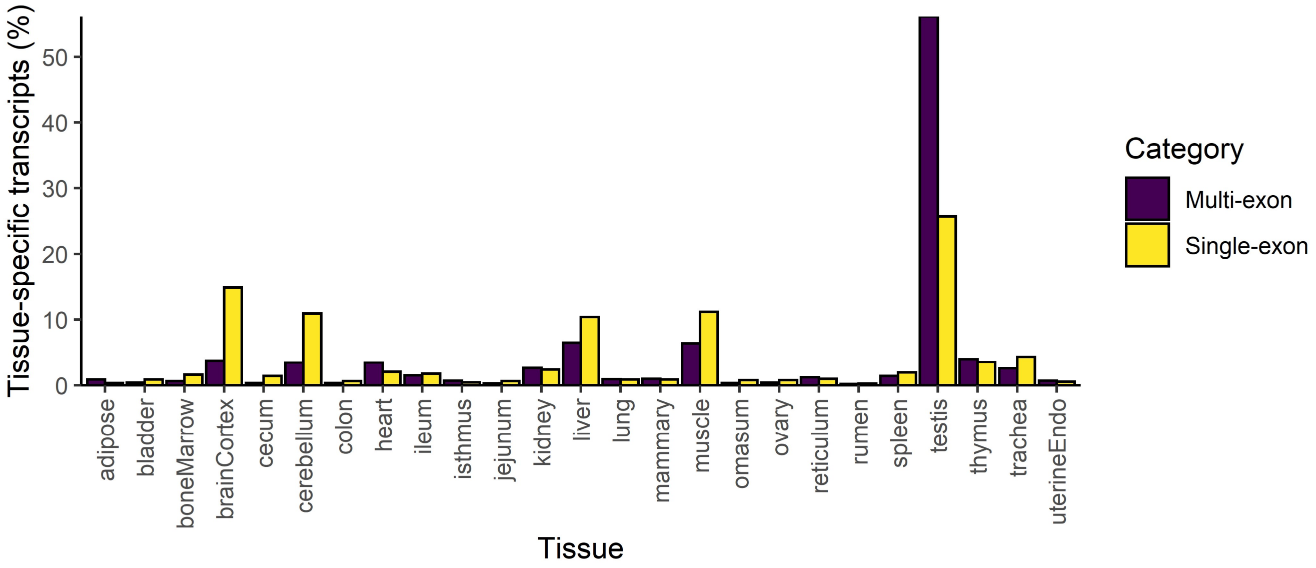

Supplementary Figure 11. Tissue-specificity of single- versus multi-exon transcripts. (A) Density of TSI scores among all predicted transcripts, multi-exon transcripts, and single-exon transcripts. (B) Percentage of single- or multi-exon tissue-specific transcripts that corresponded to each tissue.

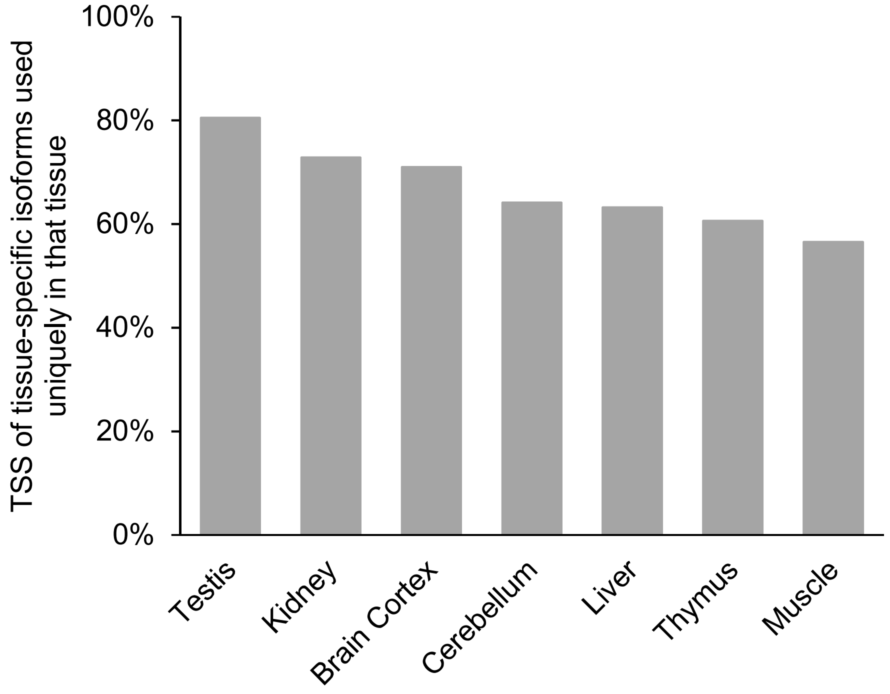

Supplementary Figure 12. Tissue-specific transcription start site usage. Proportion of TSS corresponding to tissue-specific isoforms that were only actively used in that tissue.

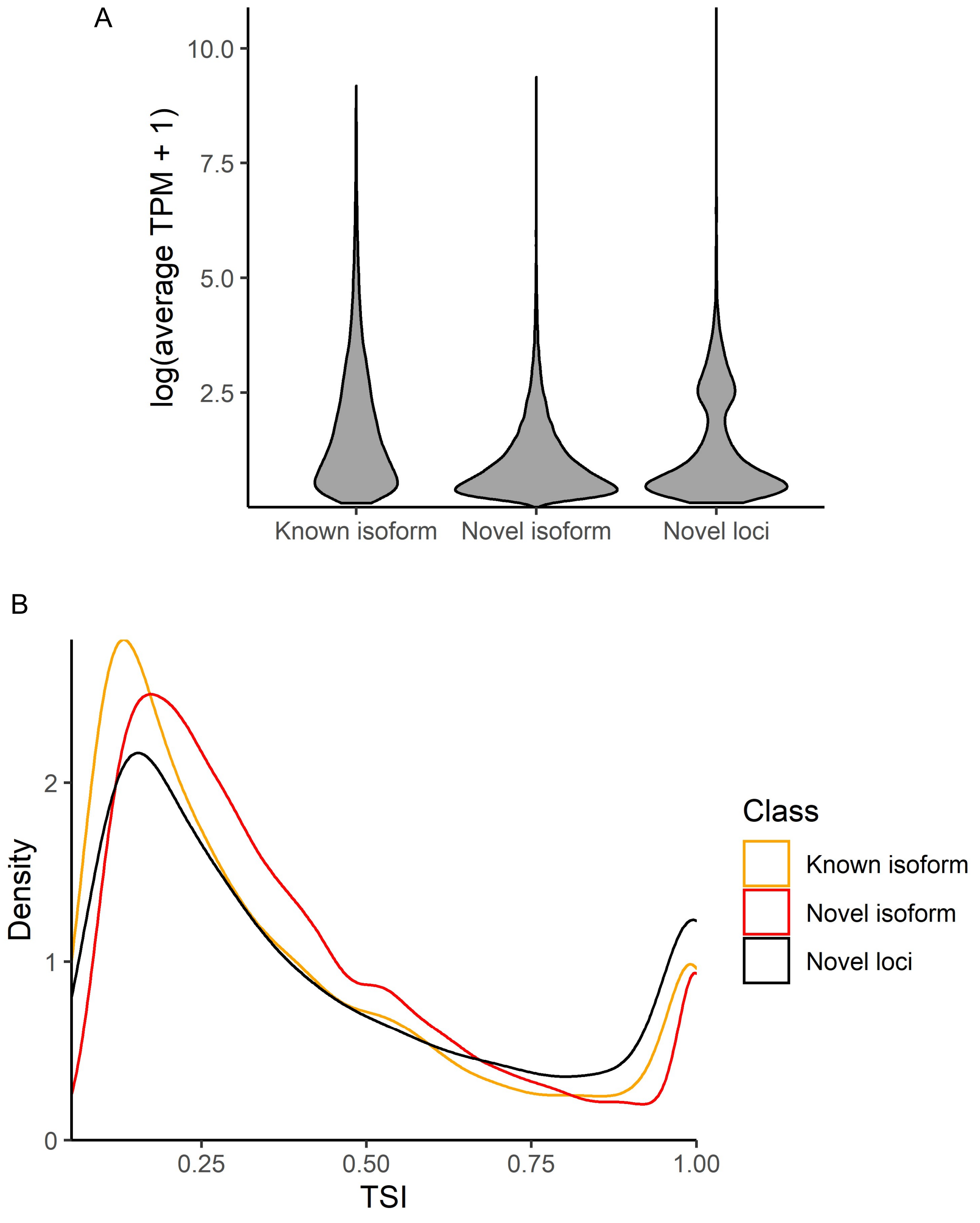

Supplementary Figure 13. Expression dynamics of novel versus reference transcript isoforms. (A) Average expression of transcripts across all samples used to identify tissue-specific transcripts, separating transcripts as either known (e.g. predicted transcript was a complete match to an annotated intron chain), novel isoform (a new transcript variant at a reference locus), or novel loci (did not correspond to an annotated locus). (B) Distribution of the TSI for known isoforms, novel isoforms, and novel loci.

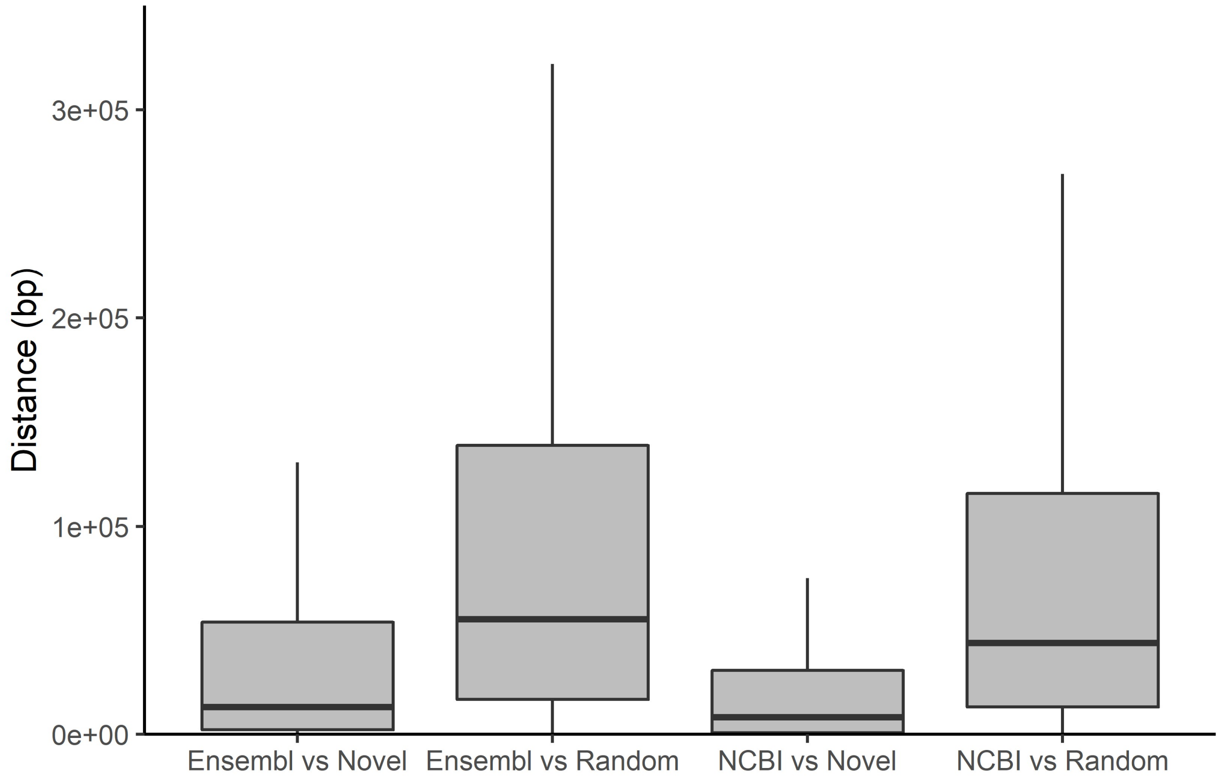

Supplementary Figure 14. Genomic distribution of novel intergenic transcripts. Distance to the nearest annotated gene (either Ensembl or NCBI) was determined for novel intergenic transcripts, and randomized genomic coordinate (excluding regions that were annotated as genes by either the Ensembl or NCBI annotations).

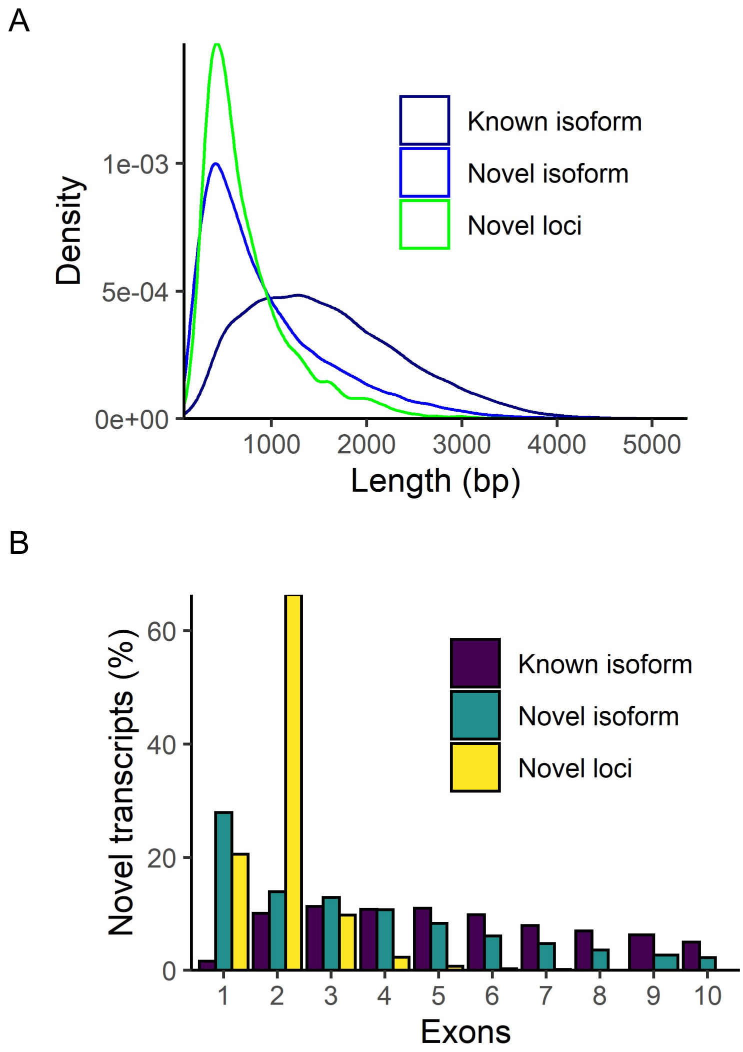

Supplementary Figure 15. Complexity of novel versus known isoforms. (A) Distribution of transcript lengths among predicted transcript classes, either previously annotated ("known") isoforms of annotated genes, novel isoforms of annotated genes, or transcripts at novel loci. (B) Proportion of transcripts, among the same classes, that had between 1 and 10 exons.

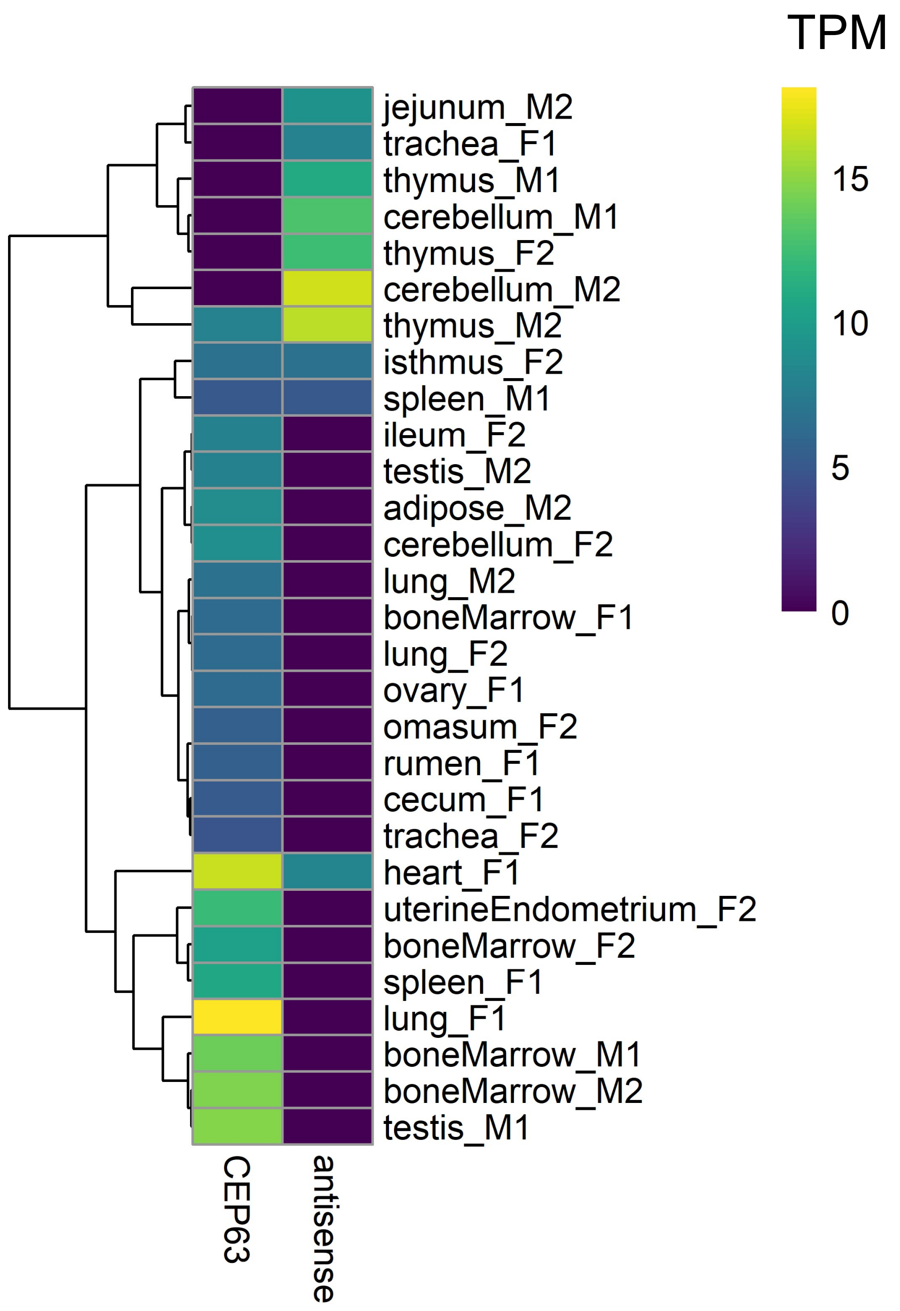

Supplementary Figure 16. Expression profiles of the most highly expressed sense *CEP63* isoform and antisense transcript. Expression is only shown for tissues with non-zero expression of at least one transcript. Expression shown in Transcripts per million (TPM).

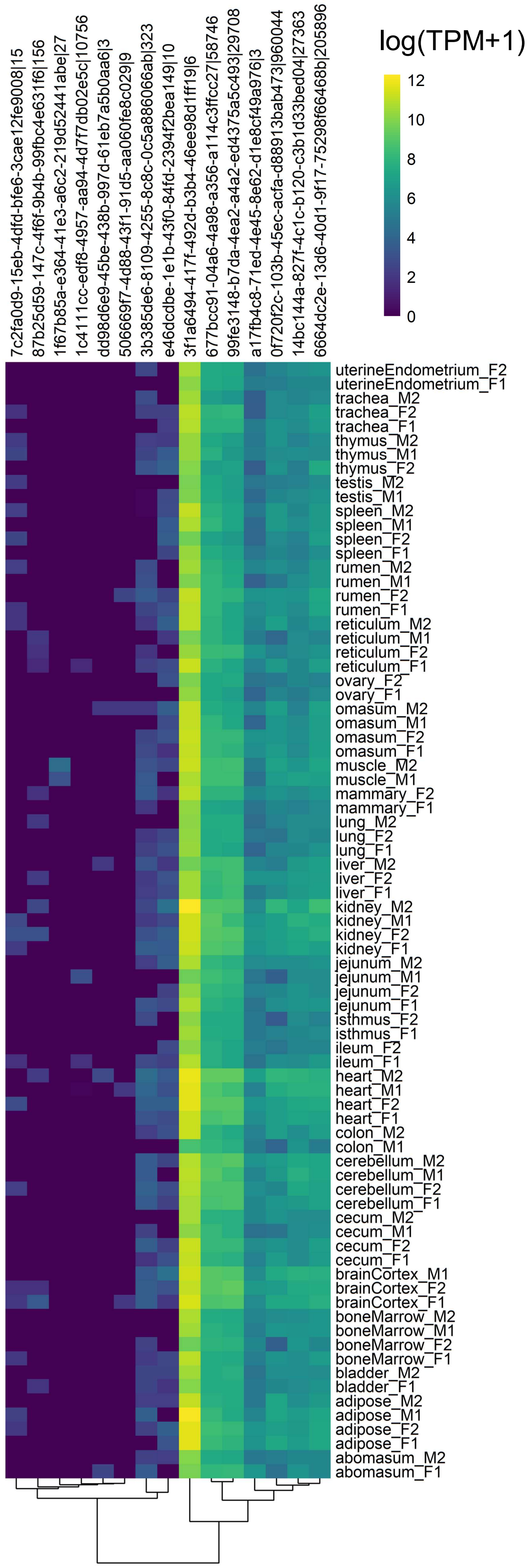

Supplementary Figure 17. Expression profiles of predicted transcripts at the chromosome 16 region from 815000-830000 bp. Expression shown as log-transformed transcripts per million (TPM).

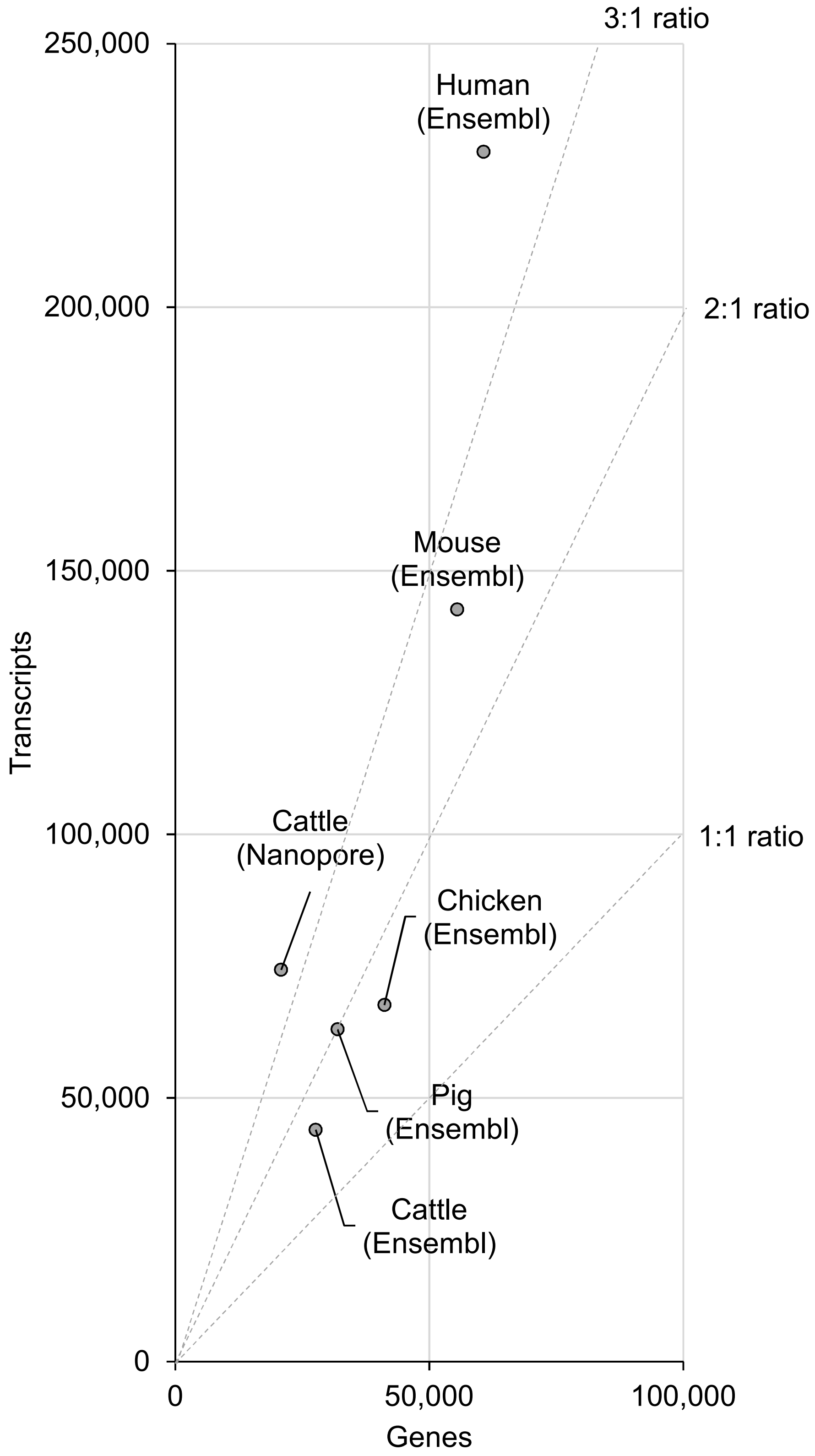

Supplementary Figure 18. Ratio of transcripts to genes in several species and annotations, including bovine transcripts predicted from ONT reads.

Supplementary Table 1. Barcode sequences associated with each sample, which were used for demultiplexing of ONT sequencing data.

Animal	Tissue	Barcode	Sequence
M1	kidney	BC01	AAGAAAGTTGTCGGTGTCTTTGTG
M1	skeletal muscle	BC02	TCGATTCCGTTTGTAGTCGTCTGT
M1	adipose	BC03	GAGTCTTGTGTCCCAGTTACCAGG
M1	spleen	BC04	TTCGGATTCTATCGTGTTTCCCTA
M1	brain cortex	BC05	CTTGTCCAGGGTTTGTGTAACCTT
M1	cerebellum	BC06	TTCTCGCAAAGGCAGAAAGTAGTC
M1	hypothalamus	BC07	GTGTTACCGTGGGAATGAATCCTT
M1	heart	BC08	TTCAGGGAACAAACCAAGTTACGT
M1	lung	BC09	AACTAGGCACAGCGAGTCTTGGTT
M1	thyroid	BC10	AAGCGTTGAAACCTTTGTCCTCTC
M1	esophagus	BC11	GTTTCATCTATCGGAGGGAATGGA
M1	thymus	BC12	CAGGTAGAAAGAAGCAGAATCGGA
M1	bone marrow	BC13	AGAACGACTTCCATACTCGTGTGA
M1	duodenum	BC14	AACGAGTCTCTTGGGACCCATAGA
M1	jejunum	BC15	AGGTCTACCTCGCTAACACCACTG
M1	cecum	BC16	CGTCAACTGACAGTGGTTCGTACT
M1	colon	BC17	ACCCTCCAGGAAAGTACCTCTGAT
M1	rumen	BC18	CCAAACCCAACAACCTAGATAGGC
M1	reticulum	BC19	GTTCCTCGTGCAGTGTCAAGAGAT
M1	omasum	BC20	TTGCGTCCTGTTACGAGAACTCAT
M1	abomasum	BC21	GAGCCTCTCATTGTCCGTTCTCTA
M1	testis	BC22	ACCACTGCCATGTATCAAAGTACG
M2	liver	BC23	CTTACTACCCAGTGAACCTCCTCG
M2	kidney	BC24	GCATAGTTCTGCATGATGGGTTAG
M2	skeletal muscle	BC25	GTAAGTTGGGTATGCAACGCAATG
M2	adipose	BC26	CATACAGCGACTACGCATTCTCAT
M2	spleen	BC27	CGACGGTTAGATTCACCTCTTACA
M2	cerebellum	BC28	TGAAACCTAAGAAGGCACCGTATC
M2	heart	BC29	CTAGACACCTTGGGTTGACAGACC
M2	lung	BC30	TCAGTGAGGATCTACTTCGACCCA
M2	trachea	BC31	TGCGTACAGCAATCAGTTACATTG
M2	bladder	BC32	CCAGTAGAAGTCCGACAACGTCAT
M2	thyroid	BC33	CAGACTTGGTACGGTTGGGTAACT
M2	esophagus	BC34	GGACGAAGAACTCAAGTCAAAGGC
M2	thymus	BC35	CTACTTACGAAGCTGAGGGACTGC
M2	skin	BC36	ATGTCCCAGTTAGAGGAGGAAACA
M2	bone marrow	BC37	GCTTGCGATTGATGCTTAGTATCA
M2	jejunum	BC38	ACCACAGGAGGACGATACAGAGAA
M2	cecum	BC39	CCACAGTGTCAACTAGAGCCTCTC
M2	colon	BC40	TAGTTTGGATGACCAAGGATAGCC
M2	rumen	BC41	GGAGTTCGTCCAGAGAAGTACACG
M2	reticulum	BC42	CTACGTGTAAGGCATACCTGCCAG
M2	omasum	BC43	CTTTCGTTGTTGACTCGACGGTAG
M2	abomasum	BC44	AGTAGAAAGGGTTCCTTCCCACTC
M2	testis	BC45	GATCCAACAGAGATGCCTTCAGTG

Animal	Tissue	Barcode	Sequence
F1	liver	BC46	GCTGTGTTCCACTTCATTCTCCTG
F1	kidney	BC47	GTGCAACTTTCCCACAGGTAGTTC
F1	adipose	BC48	CATCTGGAACGTGGTACACCTGTA
F1	spleen	BC49	ACTGGTGCAGCTTTGAACATCTAG
F1	brain cortex	BC50	ATGGACTTTGGTAACTTCCTGCGT
F1	cerebellum	BC51	GTTGAATGAGCCTACTGGGTCCTC
F1	heart	BC52	TGAGAGACAAGATTGTTCGTGGAC
F1	lung	BC53	AGATTCAGACCGTCTCATGCAAAG
F1	trachea	BC54	CAAGAGCTTTGACTAAGGAGCATG
F1	bladder	BC55	TGGAAGATGAGACCCTGATCTACG
F1	esophagus	BC56	TCACTACTCAACAGGTGGCATGAA
F1	skin	BC57	GCTAGGTCAATCTCCTTCGGAAGT
F1	mammary gland	BC58	CAGGTTACTCCTCCGTGAGTCTGA
F1	ovary	BC59	TCAATCAAGAAGGGAAAGCAAGGT
F1	isthmus	BC60	CATGTTCAACCAAGGCTTCTATGG
F1	uterine myometrium	BC61	AGAGGGTACTATGTGCCTCAGCAC
F1	uterine endometrium	BC62	CACCCACACTTACTTCAGGACGTA
F1	bone marrow	BC63	TTCTGAAGTTCCTGGGTCTTGAAC
F1	jejunum	BC64	GACAGACACCGTTCATCGACTTTC
F1	ileum	BC65	TTCTCAGTCTTCCTCCAGACAAGG
F1	cecum	BC66	CCGATCCTTGTGGCTTCTAACTTC
F1	colon	BC67	GTTTGTCATACTCGTGTGCTCACC
F1	rumen	BC68	GAATCTAAGCAAACACGAAGGTGG
F1	reticulum	BC69	TACAGTCCGAGCCTCATGTGATCT
F1	omasum	BC70	ACCGAGATCCTACGAATGGAGTGT
F1	abomasum	BC71	CCTGGGAGCATCAGGTAGTAACAG
F2	liver	BC72	TAGCTGACTGTCTTCCATACCGAC
F2	kidney	BC73	AAGAAACAGGATGACAGAACCCTC
F2	adipose	BC74	TACAAGCATCCCAACACTTCCACT
F2	spleen	BC75	GACCATTGTGATGAACCCTGTTGT
F2	brain cortex	BC76	ATGCTTGTTACATCAACCCTGGAC
F2	cerebellum	BC77	CGACCTGTTTCTCAGGGATACAAC
F2	heart	BC78	AACAACCGAACCTTTGAATCAGAA
F2	lung	BC79	TCTCGGAGATAGTTCTCACTGCTG
F2	trachea	BC80	CGGATGAACATAGGATAGCGATTC
F2	esophagus	BC81	CCTCATCTTGTGAAGTTGTTTCGG
F2	thymus	BC82	ACGGTATGTCGAGTTCCAGGACTA
F2	mammary gland	BC83	TGGCTTGATCTAGGTAAGGTCGAA
F2	ovary	BC84	GTAGTGGACCTAGAACCTGTGCCA
F2	isthmus	BC85	AACGGAGGAGTTAGTTGGATGATC
F2	uterine endometrium	BC86	AGGTGATCCCAACAAGCGTAAGTA
F2	bone marrow	BC87	TACATGCTCCTGTTGTTAGGGAGG
F2	jejunum	BC88	TCTTCTACTACCGATCCGAAGCAG
F2	ileum	BC89	ACAGCATCAATGTTTGGCTAGTTG
F2	cecum	BC90	GATGTAGAGGGTACGGTTTGAGGC
F2	rumen	BC91	GGCTCCATAGGAACTCACGCTACT
F2	reticulum	BC92	TTGTGAGTGGAAAGATACAGGACC
F2	omasum	BC93	AGTTTCCATCACTTCAGACTTGGG

Supplementary Table 2. Summary of cattle sequencing data by tissue and replicate (males: M1 and M2, females: F1 and F2), including raw reads, full-length strand-oriented reads, and uniquely mapped reads.

Tissue	Replicate	Raw reads	Full-length reads	Raw reads that were full-length (%)	Uniquely mapped full-length reads	Full-length reads that were uniquely mapped (%)
Abomasum	F1	308,851	270,841	87.69	241,528	89.18
Abomasum	M1	231,959	200,071	86.25	164,768	82.35
Abomasum	M2	213,721	186,835	87.42	167,921	89.88
Adipose	F1	301,029	261,383	86.83	223,093	85.35
Adipose	F2	396,260	334,665	84.46	282,855	84.52
Adipose	M1	310,481	265,210	85.42	227,634	85.83
Adipose	M2	338,657	291,227	85.99	245,932	84.45
Bladder	F1	515,263	445,257	86.41	367,166	82.46
Bladder	M2	288,092	247,079	85.76	205,059	82.99
Bone Marrow	F1	510,389	438,630	85.94	372,448	84.91
Bone Marrow	F2	269,573	229,699	85.21	190,376	82.88
Bone Marrow	M1	226,020	191,181	84.59	159,290	83.32
Bone Marrow	M2	213,371	183,475	85.99	154,032	83.95
Brain Cortex	F1	450,374	381,654	84.74	335,135	87.81
Brain Cortex	M1	408,826	353,540	86.48	312,378	88.36
Brain Cortex	F2	638,907	554,653	86.81	472,262	85.15
Cecum	F1	561,305	488,420	87.02	411,818	84.32
Cecum	F2	298,580	257,178	86.13	215,504	83.80
Cecum	M1	334,458	292,627	87.49	232,618	79.49
Cecum	M2	285,201	246,357	86.38	209,179	84.91
Cerebellum	F1	355,617	300,654	84.54	267,060	88.83
Cerebellum	F2	370,090	314,069	84.86	272,040	86.62
Cerebellum	M1	592,479	505,628	85.34	457,274	90.44
Cerebellum	M2	605,949	521,109	86.00	463,032	88.86
Colon	F1	392,996	339,772	86.46	289,596	85.23
Colon	M1	295,789	261,807	88.51	215,641	82.37
Colon	M2	303,813	267,257	87.97	224,990	84.18
Duodenum	M1	95,565	78,690	82.34	61,768	78.50
Esophagus	F1	741,662	650,998	87.78	546,207	83.90
Esophagus	F2	415,177	358,296	86.30	304,017	84.85
Esophagus	M1	389,615	337,931	86.73	291,421	86.24
Esophagus	M2	286,199	246,711	86.20	203,709	82.57
Heart	F1	293,000	250,367	85.45	218,587	87.31
Heart	F2	333,583	277,383	83.15	238,147	85.85
Heart	M1	431,897	367,137	85.01	319,157	86.93
Heart	M2	400,402	341,010	85.17	297,543	87.25
Hypothalamus	M1	362,545	311,447	85.91	274,218	88.05
Ileum	F1	631,048	541,043	85.74	440,374	81.39
Ileum	F2	383,203	329,001	85.86	265,419	80.67
Isthmus	F1	358,784	311,359	86.78	260,785	83.76
Isthmus	F2	454,593	388,021	85.36	312,573	80.56
Jejunum	F1	342,487	295,579	86.30	247,160	83.62
Jejunum	F2	296,544	249,317	84.07	205,144	82.28
Jejunum	M1	150,258	125,865	83.77	98,734	78.44
Jejunum	M2	302,025	263,656	87.30	220,664	83.69
Kidney	F1	366,184	315,857	86.26	272,851	86.38
Kidney	F2	302,170	256,555	84.90	216,985	84.58
Kidney	M1	357,591	306,302	85.66	264,542	86.37
Kidney	M2	259,584	228,904	88.18	188,102	82.18
Liver	F1	318,227	273,400	85.91	237,509	86.87
Liver	F2	332,431	286,426	86.16	247,607	86.45
Liver	M2	415,309	360,051	86.69	307,137	85.30
Lung	F1	347,285	299,759	86.31	253,875	84.69
Lung	F2	463,079	394,782	85.25	320,951	81.30
Lung	M1	1,561	1,201	76.94	953	79.35
Lung	M2	452,752	392,375	86.66	318,603	81.20
Mammary	F1	410,589	358,378	87.28	297,621	83.05
Mammary	F2	643,647	563,283	87.51	468,797	83.23
Muscle	M1	460,616	393,197	85.36	348,293	88.58
Muscle	M2	373,504	313,772	84.01	272,293	86.78
Omasum	F1	557,186	470,301	84.41	383,203	81.48
Omasum	F2	512,319	437,825	85.46	361,714	82.62
Omasum	M1	367,445	320,297	87.17	251,272	78.45
Omasum	M2	379,623	328,518	86.54	268,154	81.63
Ovary	F1	480,422	413,119	85.99	333,671	80.77
Ovary	F2	317,019	271,630	85.68	217,226	79.97
Reticulum	F1	809,966	684,602	84.52	571,486	83.48
Reticulum	F2	520,304	444,784	85.49	372,116	83.66
Reticulum	M1	416,782	358,951	86.12	299,321	83.39
Reticulum	M2	425,210	367,300	86.38	313,190	85.27
Rumen	F1	497,108	419,223	84.33	342,054	81.59
Rumen	F2	262,461	222,060	84.61	178,547	80.40
Rumen	M1	438,953	380,135	86.60	309,411	81.40
Rumen	M2	325,132	273,146	84.01	224,632	82.24
Skin	F1	310,439	268,897	86.62	229,718	85.43
Skin	M2	308,684	267,029	86.51	223,091	83.55
Spleen	F1	286,118	245,855	85.93	201,182	81.83
Spleen	F2	264,207	216,802	82.06	169,008	77.96
Spleen	M1	655,512	568,114	86.67	471,297	82.96
Spleen	M2	396,661	346,154	87.27	281,222	81.24
Testis	M1	405,973	344,295	84.81	307,180	89.22
Testis	M2	357,197	304,977	85.38	273,942	89.82
Thymus	F2	262,447	222,852	84.91	169,944	76.26
Thymus	M1	293,263	251,547	85.78	212,977	84.67
Thymus	M2	386,367	338,187	87.53	287,002	84.86
Thyroid	M1	267,802	231,708	86.52	191,665	82.72
Thyroid	M2	332,034	285,645	86.03	225,434	78.92
Trachea	F1	365,991	314,672	85.98	265,451	84.36
Trachea	F2	666,078	583,324	87.58	479,278	82.16
Trachea	M2	444,601	386,811	87.00	340,126	87.93
Uterine Endometrium	F1	285,331	244,272	85.61	188,875	77.32
Uterine Myometrium	F2	258,527	221,772	85.78	178,591	80.53
Uterine Myometrium	F1	219,650	189,533	86.29	148,638	78.42
Total	--	*35,267,978*	*30,322,668*	--	*25,472,993*	--
Average	--	*379,226*	*326,050*	*85.98*	*273,903*	*84.01*

Supplementary Table 3. Summary of uniquely mapped full-length reads by tissue and sample, showing average reads obtained per tissue.

	Uniquely mapped full-length reads				
	Female 1	Female 2	Male 1	Male 2	*Total*
Abomasum	241,528	--	164,768	167,921	*574,217*
Adipose	223,093	282,855	227,634	245,932	*979,514*
Bladder	367,166	--	--	205,059	*572,225*
Bone marrow	372,448	190,376	159,290	154,032	*876,146*
Brain cortex	335,135	472,262	312,378		*1,119,775*
Cecum	411,818	215,504	232,618	209,179	*1,069,119*
Cerebellum	267,060	272,040	457,274	463,032	*1,459,406*
Colon	289,596	--	215,641	224,990	*730,227*
Duodenum	--	--	61,768		*61,768*
Esophagus	546,207	304,017	291,421	203,709	*1,345,354*
Heart	218,587	238,147	319,157	297,543	*1,073,434*
Hypothalamus	--	--	274,218	--	*274,218*
Ileum	440,374	265,419	--	--	*705,793*
Isthmus	260,785	312,573	--	--	*573,358*
Jejunum	247,160	205,144	98,734	220,664	*771,702*
Kidney	272,851	216,985	264,542	188,102	*942,480*
Liver	237,509	247,607	--	307,137	*792,253*
Lung	253,875	320,951	953	318,603	*894,382*
Mammary gland	297,621	468,797	--	--	*766,418*
Skeletal muscle	--	--	348,293	272,293	*620,586*
Omasum	383,203	361,714	251,272	268,154	*1,264,343*
Ovary	333,671	217,226	--	--	*550,897*
Reticulum	571,486	372,116	299,321	313,190	*1,556,113*
Rumen	342,054	178,547	309,411	224,632	*1,054,644*
Skin	229,718	--	--	223,091	*452,809*
Spleen	201,182	169,008	471,297	281,222	*1,122,709*
Testis	--	--	307,180	273,942	*581,122*
Thymus	--	169,944	212,977	287,002	*669,923*
Thyroid	--	--	191,665	225,434	*417,099*
Trachea	265,451	479,278	--	340,126	*1,084,855*
Uterine endometrium	188,875	178,591	--	--	*367,466*
Uterine myometrium	148,638	--	--	--	*148,638*
				Average	*796,031*
